# *Ex uno, multis*: taxonomic revision in *Navarretia divaricata* (Polemoniaceae) and the recognition of four additional cryptic or near-cryptic species

**DOI:** 10.3897/phytokeys.91.21530

**Published:** 2017-12-05

**Authors:** Leigh A. Johnson, David Gowen

**Affiliations:** 1 Department of Biology and M.L. Bean Life Science Museum, Brigham Young University, Provo, Utah 84602, USA; 2 111 Roble Road, Oakland, California 94618, USA

**Keywords:** allopolyploidy, California, conservation assessment, exclusivity, species delimitation, taxonomy, unified species concept, western United States

## Abstract

*Navarretia divaricata*, endemic to western North America and most recently considered a single species with two subspecies, was re-examined in light of field work, DNA sequences, comparative morphology, and a review of herbarium specimens including types. From these studies, we lectotypify the material on which *N. divaricata* is based, elevate N. divaricata subsp. vividior, which is an allotetraploid, to species rank (as *N. vividior* **comb. et stat. nov**.), and recognize three additional species: *N. modocensis* **sp. nov.**, *N. aeroides* **sp. nov.**, and *N. torreyella* **sp. nov.** *Navarretia modocensis*, the diploid paternal progenitor of *N. vividior*, is morphologically cryptic with respect to its allotetraploid offspring and difficult to distinguish on herbarium sheets. *Navarretia aeroides*, the diploid maternal progenitor of *N. vividior*, is nearly cryptic, but more easily distinguished from both *N. modocensis* and *N. vividior* by its smaller, more glandular inflorescences. *Navarretia torreyella* is readily distinguished from all of these species, but has been generally mistaken for N. divaricata subsp. vividior given its colored corolla tube and rare co-occurrence with the other *vividior*-like species. Conservation assessments, an identification key, and table of comparative morphological features are provided for each species, emended descriptions for *N. divaricata* and *N. vividior*, and a discussion of the syntypes for *Gilia divaricata* Torr. ex A.Gray.

## Introduction

Multiple criteria applied to assess the presence of homogenizing gene flow under the framework of the unified species concept (de Queiroz 2007) has provided strong evidence for the existence of previously unrecognized lineages in Polemoniaceae, including in *Navarretia* (Johnson and Cairns-Heath 2010, Johnson et al. 2012, 2013, 2016). Overlooked in the past because they resemble other species, re-examination of morphological variation in conjunction with molecular data has enabled these cryptic or nearly cryptic lineages to be delineated and lifted from obscurity as recognized taxa. In some cases, molecular data provided the first clues as to the uniqueness of these taxa (e.g., Johnson and Cairns-Heath 2010, Johnson et al. 2012), whereas in other cases, morphological variation was recognized first, and the biological significance of this variation confirmed with molecular data (Johnson et al. 2013, 2016). Stimulated by a need for nomenclatural housekeeping along with observations of variation in both morphological and molecular data, we here delimit several near-cryptic species from what previously has been considered a single species, *Navarretia divaricata* Greene.

Within *Navarretia* Ruiz & Pav., *N. divaricata* is one of the more widely distributed species with populations extending from central California (and adjacent Nevada) to Idaho and British Columbia. It is also one of the smallest-flowered *Navarretia*. The taxonomic concept associated with *N. divaricata* has been fairly stable since Greene’s treatment of the species in this genus (Greene 1887), and Jepson’s subsequent partitioning of “coarser” specimens with more glandular-puberulent bracts and calyces, and blue or blue-lobed corollas into variety *vividior* Jeps. & V.L.Bailey (in Jepson 1943; currently subsp. vividior (Jeps. & V.L.Bailey) H.Mason). To preserve this stability, clarification of the type for *N. divaricata* Greene and lectotypification of the basionym upon which Greene’s replacement name is based is necessary. Furthermore, stemming from observations that N. divaricata subsp. divaricata does not form a monophyletic group with N. divaricata subsp. vividior in comparative DNA sequence analyses (Johnson et al. 2016), that the two taxa co-occur without signs of hybridization, and that material assigned to N. divaricata subsp. vividior is polymorphic, we initiated fieldwork, a comprehensive review of herbarium specimens, and investigation of morphological and molecular variation across the geographic ranges of these taxa. Results indicate that N. divaricata subsp. vividior merits recognition at the species level, and that four evolutionarily unique lineages exist in the material heretofore generally referred to subsp. vividior. The taxonomy of this group is revised accordingly.

### Historical background

Among plants provided to Asa Gray by John Torrey, two *Navarretia* collections are relevant to the present study. The first, labeled simply “*Navarretia*” by Torrey, was collected during his 1865 trip to California. The second, collected by Mr. Shelton, was labeled by Torrey, “*Navarretia divaricata*, n. sp.” Gray considered these specimens conspecific, but having a confused view of generic relationships in Polemoniaceae (Mason 1945), he published this new species, attributed to Torrey, in the genus *Gilia* (i.e., *Gilia divaricata* Torr. ex A.Gray) where *Gilia divaricata* Nutt. (≡ *Allophyllum divaricatum* (Nutt.) A.D.Grant & V.E.Grant) already existed. Consequently, Gray’s action created an illegitimate later homonym that was replaced by Greene (1887). In the protologue, Gray (1870) states, “California, along the foot hills of the Sierra Nevada, coll. Shelton, Rattan, Bolander, Torrey, Mrs. Davis, C. Lee,” providing four additional syntypes beyond the two that can be traced to Torrey. The last of these, a specimen attributed to C. Lee, cannot be located and its identity remains unknown to us. The remaining five syntypes represent four distinct taxa. Consequently, the choice of lectotype is critical for maintaining the long-used taxonomic concept for *N. divaricata*.

Gray (1876, 1878) continued to recognize *Gilia divaricata* Torr., using descriptions similar to his original. These descriptions accentuate characteristics of the syntype *Rattan s.n.* (NY!; probable duplicates at UC × 2 (scan!), US (scan!), KEW (scan!)), which has larger flowers and a more densely pubescent head compared to the other syntypes, and is also a mixed collection from two gatherings. These specimens are today recognized as *N. prolifera* Greene, which has the narrowest distribution of the taxa represented by Gray’s syntypes.

Emphasizing the distinctiveness of the calyx in *Navarretia*, Greene (1887) began the work of narrowing Gray’s concept of *Gilia* and provided a replacement name for Gray’s illegitimate homonym by equating *N. divaricata* Greene with *Gilia divaricata* Torr. ex Gray. There is no indication that Greene viewed the syntypes Gray worked with, and good reason to believe he did not—for example, he described *N. prolifera* as a new species immediately preceding *N. divaricata*, with no reference to any of the *Gilia divaricata* Torr. ex Gray syntypes. Greene characterized *N. divaricata* flowers as “minute” and distributed in the Sierra Nevada from Kern County northward into Oregon; he distinguished this taxon from *N. prolifera* and *N. peninsularis* Greene in part based on smaller flower size. Beyond the description provided for *N. divaricata*, Greene’s taxonomic concept can be reconstructed by examining the specimens he worked with. Four sheets with five collections exist at NDG (scans!) labeled “*Navarretia divaricata* Greene”, in Greene’s hand, with collection dates prior to Greene’s publication, and a sixth specimen, also on one of the four sheets, with a preprinted label collected in the year of his publication, labeled “*Gilia divaricata* Torr.” Of these, Greene referenced two in his publication.

*M. K. Curran s.n*. [NDG-40817], from Tehachapi, Kern County, California, was cited as representing the southern extent of the species range, but differs taxonomically from the other specimens and is equivalent to *N. peninsularis*. Interestingly, this collection represents the northern extent of the latter species rather than the southern extent of *N. divaricata*. Greene described *N. peninsularis* based on larger plants from Lower (Baja) California, considerably distant from Tehachapi, so perhaps the Tehachapi plants being more depauperate and proximal to other *N. divaricata* specimens played a role in his misidentification and efforts to circumscribe taxa with little material on hand. The second specimen, *C. F. Sonne s.n.* (Donner Lake) [NDG-40819A], was cited as a putative hybrid between *N. divaricata* and *N. minima* Nutt. without explanation. This specimen, along with *C. C. Parry s.n.*, (Yosemite) [NDG-40820A], *D. Cleveland s.n.* (Butte County) [NDG-40820B], and *T. Howell s.n*. (near Waldo, Oregon) [NDG-40819B] all correspond to material congruent with Gray’s syntypes *Bolander 4908* and *Mrs. Davis 49*, while a second *M. K. Curran s.n.* specimen, from Colusa County, California [NDG-40818], is congruent with Gray’s syntype *Torrey 302* discussed further below. Gray’s syntypes *Bolander 4908* and *Mrs. Davis 49* have minute flowers and the species represented by them is the most common and widely distributed geographically of Gray’s syntypes, as well as the entity to which the name N. divaricata subsp. divaricata has been applied.

Greene’s taxonomic concept influenced nearly all later botanists. For example, Howell (1901) indicated *N. divaricata* occurs from “Washington to California, in the high mountains,” with a description that borrowed more from Greene’s description of the species than Gray’s. Though referencing neither Gray nor Greene, the publication of *Gilia atrata* M.E.Jones with its type and two of three paratypes matching *Bolander 4908* and *Mrs. Davis 49* (and the third a similarly small-flowered entity that would fit within Greene’s concept) may stem from Jones’ (1908) recognition that Gray’s name was illegitimate while disagreeing with Greene in recognizing *Navarretia* as distinct from *Gilia. Gilia atrata* has not appeared in print, other than as a synonym, beyond its original publication.

The syntype *Torrey 302* was gathered in Lake County, California, well removed from the Sierra Nevada. Circa 1936, Virginia Bailey began a study of *N. divaricata* and noticed four specimens from Butte, Lake, and Mendocino counties that were stouter of habit, slightly larger flowered, produced more seeds per locule, and possessed blue corollas or corolla lobes (note attached to *Austin 827*, UC!). *Torrey 302*, though not seen by her, fits naturally into the group she was segregating. Jepson (1943) with Bailey, recognized this material as a variety of *N. divaricata* and chose a specimen also from Lake County as the type. Jepson and Bailey’s taxonomic concept for the two varieties of *N. divaricata* is clear, with all 17 representative specimens listed for N. divaricata var. divaricata morphologically congruent with *Bolander 4908* and *Mrs. Davis 49*, and nine representative specimens listed for var. vividior (paratypes) that all share the larger flowering heads, more robust habit, and larger/more glandular heads as described by Jepson and Bailey. Thus, in recognizing var. vividior, Jepson and Bailey followed a taxonomic concept for the typical variety of *N. divaricata* that matches the syntypes *Bolander 4908* and *Mrs. Davis 49*, despite not examining these syntypes or making reference to them. Mason (1951) treated these varieties as subspecies, but otherwise did nothing to alter the taxonomic concept for them. The description by Cronquist et al. (1984) of *N. divaricata* includes features, such as unequal valves and two of the three stigmas almost entirely fused, that exist only in material congruent with var. divaricata as put forth by Jepson and Bailey, and congruent with the syntypes *Bolander 4908* and *Mrs. Davis 49*.

The remaining syntype, *Mr. Shelton s.n*. (NY!), is distinct morphologically from the other syntypes. It has smaller heads and slender branches, like *N. divaricata subsp. divaricata*, yet has a purple corolla throat and tube, three equal valves, and three equal stigma lobes that readily separates it from that taxon. While material matching this syntype was available to Jepson and Bailey, none of those collections were included in the list of representative specimens for either var. divaricata or var. vividior. No locality information is provided for Mr. Shelton’s collection, though the material that it matches occurs in only a portion of the Sierra Nevada, with known occurrences in close proximity to Sacramento where he lived (Ewan 1981). Mr. Shelton’s specimen is of particular interest because it is the only syntype labeled “*Navarretia divaricata* n. sp.” in Torrey’s hand. An unpublished annotation attached to the sheet at NY, upon which *Mr. Shelton s.n*. and three of the remaining five syntypes are mounted, suggests Shelton’s collection is the lectotype—attributing the first step to Jepson (1943) and the second step to Cronquist (1984). We, however, disagree with the inferences made by this annotation. Jepson did not specifically state he was indicating the type, but instead restated the type locality published by Gray with his exact words reading, “type loc. ‘foothills of the Sierra Nevada,’ Shelton (the first named collector).” This falls short of specifying the Shelton collection as the lectotype, particularly since there is no indication on the sheet that the Shelton collection comes from the Sierra Nevada (even though we have determined, based on morphology, that it does). Jepson (1943) elsewhere explicitly indicates his observation of types, such as the isotype for *N. peninsularis*, but no such indication is provided for *N. divaricata*. It is unlikely Jepson would have specified as lectotype a specimen that varied from the concept he and Bailey defined through extensive specimen citations. Cronquist et al.’s actions also do not constitute a second step lectotypification. The treatment of *N. divaricata* in this work states “Shelton s.n., Sierra Nevada, Calif., is the first specimen cited by Gray, and the only one annotated by Torrey as ‘n. sp.’; isotype at NY!” Importantly, the citation above is not an act of lectotypification; the first volume of the *Intermountain Flora* expressly states, “When one of several collections cited in the protologue is obviously the primary basis for a name, we have given it as the type, without further comment. Sometimes the typification is less clear and a more cautious statement is necessary. Our citation is not to be taken as the formal selection of a lectotype, except when specifically so indicated” (Cronquist et al. 1972: 8).

To summarize, for over 125 years, the name *Navarretia divaricata* has been equated with a divaricately branched plant with minute flowers and branches that often arise immediately below a flowering head. Since 1943, the typical variety or subspecies has been associated with specimens having whitish flowers that dry with pink lobes, and a whitish to yellowish lower throat (sometimes streaked with red) and upper tube. Such plants are represented among the syntypes of *Gilia divaricata* Torr. ex Gray by *Bolander 4908* and *Mrs. Davis 49*. Choosing either *Rattan s.n.* (based on Gray’s original description, though multiple gatherings is problematic) or *Mr. Shelton s.n.* (based on Torrey’s designation) as lectotype would result in needless and confusing nomenclatural shuffling for one of the most recognizable and geographically widespread *Navarretia* species. Similarly, considering *N. divaricata* Greene to represent a new name with Curran’s cited specimen as type, independent of *G. divaricata* Torr. ex A.Gray (which was clearly not Greene’s intent), would make *N. divaricata* synonymous with *N. peninsularis*. This action would necessitate a new combination for the entity under consideration based on *Gilia atrata* M.E.Jones, a name buried in obscurity and unused since its original publication. As allowed by the code, we advocate the continued interpretation of Greene’s name as a replacement for Gray’s, with Gray’s syntypes as the original material, and designate one of these syntypes as lectotype to serve as the basionym of *N. divaricata* Greene.

## Taxonomic treatment

### Navarretia divaricata

Taxon classificationPlantaeEricalesPolemoniaceae

Greene, Pittonia 1(8): 136. 1887 emend. L.A.Johnson & D.Gowen

Figs 1 , 2

Gilia divaricata Torr. ex A.Gray, Proc. Amer. Acad. Arts 8: 270. 1870, non Gilia divaricata Nutt. 1848.
Type: United States of America. California: Mariposa County, Yosemite trail, 1866, *Bolander 4908* (lectotype, designated here: GH scan! [GH-00274983]; isolectotypes: UC scan! [UC-23512], YALE scan! [YU-065402], US scan! [US-322192], KEW, middle of three specimens, flanked on either side by *Rattan s.n*, scan! [K-000769074]). Replaced synonym.
Gilia atrata M.E.Jones, Contr. W. Botany 12: 55. 1908.
Type: United States of America. Idaho: Washington County, Salmon Meadows, 4000 ft, 22 July 1899, *Jones s.n.* (holotype: POM, top three specimens on sheet, scan! [POM-75127]).
Navarretia prolifera Greene var. breviflora M.Peck, Proc. Biol. Soc. Washington 50(24): 94. 1937.
Type: United States of America. Oregon: Douglas County, Diamond Lake, 3 July 1936, *Peck 19234* (holotype: WILLU! [WILLU-18416]; isotype: OSC scan! [OSC-43592]).

#### Type.

Based on *Gilia divaricata* Torr. ex A.Gray, non *Gilia divaricata* Nutt. 1848.

#### Emended description.

***Taprooted annual herbs*** to 15 cm tall and 20 cm wide, sometimes larger, often wider than tall. Primary stem erect, terminating in an inflorescence head 1–5 cm above the cotyledons; generally greatly exceeded by secondary stems, with tertiary, and quaternary stems present on larger plants (these higher order stems may be reduced in length and the inflorescence heads ± congested); higher order branches arise from axils of proximal inflorescence bracts, axils of leaves subtending the primary head, or less commonly, leaves within 1 cm of an inflorescence head; branches ascending to spreading and ± leafless, except for leaves subtending higher order branches or within 1 cm of a head; stem and branches tan to reddish-brown, glabrous or sparsely minutely glandular pubescent to glabrescent, less often villous, the trichomes generally less than 0.5 mm long. Cotyledons two, linear, entire, united at base. ***Leaves*** somewhat finely stipitate-glandular proximally, less so distally; leaves at the lowermost nodes opposite, linear-filiform, and widened at the point of stem attachment, the proximal nodes often congested with overlapping leaf bases. More distal leaves alternate, entire, or more commonly with 1–3 paired or unpaired linear lateral lobes 1–5 mm long attached along the proximal 3(–15) mm of the leaf, with an elongated, linear terminal segment. ***Inflorescences*** head-like, generally ≤ 10 mm diameter (exclusive of bract tips; ~15 mm with bract tips), mostly less than 15 flowered, villous proximally, obscurely glandular. Inflorescence bracts < 10(–13) mm long, ± palmatifid to subpalmatifid; outermost 1–2 bracts with a short achlorophyllous base and 2–3 pairs of lateral lobes flanking an elongate terminal lobe, the distal pair of lateral lobes sometimes shorter and reflexed somewhat out of plane relative to the other lobes; bract bases become larger and clasping centripetally with lateral lobes reduced to a single pair departing from near the apex of the bract base flanking the central terminal lobe, all bract lobes chlorophyllous, entire, long tapering acute. Bracts sparsely villous abaxially, more densely villous adaxially and proximally along the lobes just above their point of attachment, with the distal 1/2 of each lobe more or less glabrous or with a few minute stipitate glands. ***Flowers*** actinomorphic, ***calyces*** mostly 4.5–7.5 mm long, tube ~ 1.5–2 mm; costae entire, long tapering acute, strongly unequal to subequal with typically two costae longer than the other three; costae narrowing proximally, the shorter ones narrower at base than the intercostal membrane and the longer ones subequal with the membrane; calyx tube achlorophyllous, minutely glandular-puberulent on the intercostal membrane with the costae at least somewhat villous, the trichomes longest along the costae at the junction with the intercostal membrane, the free portion of the costae glabrous to very sparsely and minutely glandular distally; intercostal membrane v-shaped at sinus. ***Corolla*** generally shorter than longest calyx costae at anthesis but exceeding the calyx as fruit matures, narrowly funnelform, glabrous, 3.5–5.0 mm long, lobes 0.6–1.0 mm long × 0.4–0.9 mm wide, proximal tube white, distal tube and throat yellow, sometimes red-streaked, transitioning to white lobes suffused with pink or lavender at tips (drying pink); tube base expanding and investing the fruit apex. ***Stamen*** filaments unequal, 0.1–0.6 mm long, inserted unequally to subequally 0.2–1.0 mm below corolla sinuses, included in throat to slightly exserted; pollen white, apertures pantoporate, acolpate; sexine seimitectate, reticulate, heterobrochate. ***Ovary*** two-chambered, unequally three-valved with two values bearing a septum and the third valve smaller and lacking a septum entirely or nearly so, stigma obscurely three-lobed, minute, unequally divided with two stigmatic lobes nearly entirely fused, generally included in the corolla throat; capsules mostly 2–2.5 mm long, dehiscing circumcisally around the base with valves splitting upward loculicidally (completely) and septicidally (partially). ***Seeds*** generally 5–9(–12) per fruit, medium brown, ovoid-angular, mucilaginous when wet. ***Nuclear gene*** loci showing diploid PCR amplification patterns.

#### Habitat, distribution, and phenology.

*Navarretia divaricata* occurs on a variety of soils from (800)1000–2600 meters in foothill and mountain habitats. It is widely distributed from Santa Barbara and Tulare Counties, California in the south to just within the borders of British Columbia, Canada, in the north, and east to Nevada and Idaho (Fig. 2). Flowering time is (May)June–July(September).

#### Conservation status.

*Navarretia divaricata* has many occurrences throughout its broad range, and is typically abundant when encountered. Occurrences near the periphery of its range (e.g., in British Columbia) may be limited in numbers, but the species is otherwise well established. It is a species of Least Concern following IUCN (2012) Red List version 3.1 criteria.

#### Etymology.

From the Latin *divaricatus*, to spread or diverge at a wide angle, which aptly describes the characteristic repeated pattern of nearly leafless stems diverging from under flowering heads to give rise to additional, well-separated flowering heads in this species and its morphological allies.

#### Representative specimens examined.

**Canada**. **British Columbia**: Anarchist Mt., 1.6 km north of Hwy 3 rest stop, 6.5 km due east of Osoyoos Lake, 49.0275°N, 119.35°W, 1350 m, 10 July 2000, *Lomer 3838* (UBC Scan); Anarchist Mt., 7 km due east of Osoyoos Lake, 1.5 km due southwest of summit, 200 m northeast of tower, 49.02658°N, 119.34786°W, 1380 m, 9 July 2013, *Lomer 8435* (UBC scan).

**United States of America.** All seventeen specimens cited by Jepson (1943) under Navarretia divaricata var. divaricata (JEPS); **California**: Alpine County, North side of Hwy 88 along an old dirt road, 38.7240°N, 119.9542°W, 2262 m, 10 July 2014, *Johnson 14-179* (BRY); Amador County, down slope from Tragedy Springs Monument, 38.6389°N, 120.1465°W, 2406 m, 8 July 2014, *Johnson 14-159* (BRY); Butte County, ca. 0.7 miles up Humboldt Rd to Butte Meadows from Hwy 32, 40.0407°N, 121.6056°W, 1204 m, 11 June 2015, *Johnson 15-045* (BRY); Calaveras County, at entrance to old lumber road off of Hwy 4 ca. 1.6 miles northeast of Camp Connell Maintenance station, 38.3356°N, 120.2278°W, 1 July 1993, *Johnson 93-112* (BRY); North side of Avery-Sheep Ranch Road ca. 2 miles from Hwy 4, 38.1969°N, 120.4003°W, 1098 m, 4 June 2014, Johnson et al. *14-068* (BRY); El Dorado County, ca. 0.1 mile west of NF-8N55 on Omo Ranch Road, 38.5542°N, 120.5401°W, 1172 m, 29 May 2013, *Johnson 13-189* (BRY); off road to Ice House Reservoir, 6.4 miles from jct. with Hwy 50, 38.7975°N, 120.4028°W, 1596 m, 30 May 2013, *Johnson 13-219* (BRY); Sly Park Vicinity, Park Creek Road 3.7 miles from county road E16, 38.7493°N, 120.4970°W, 1207 m, 8 July 2014, *Johnson 14-141* (BRY); Lassen County, west of county road A21along dirt road leading to large moist swell, 40.3601°N, 121.0039°W, 1589 m, 2 August 2006, *Johnson 06-129* (BRY); Nevada County, 39.32°N, 120.75°W, 1565 m, 3 June 2014, *Johnson* et al. *14-018* (BRY); Placer County, Off Sawtooth Ridge Rd ca. 1.1 mile west of Dawson Spring, 39.21787°N, 120.6226°W, 1670 m, 3 June 2014, *Johnson* et al. *14-029* (BRY); Plumus County, south of Cascade and Lava Top, access up FS road 21N22YA, 39.6827°N, 121.1665°W, 1402 m, 10 June 2015, *Johnson & Ahart 15-024* (BRY); Tehama County, two miles south of Hwy 36/89 on Hwy 32, 40.2661°N, 121.4509°W, 4590 ft, *Johnson 04-140* (BRY); Shasta County, off Hwy 89 ca. 1.3 miles north of jct with Hwy A19, 41.1612°N, 121.6609°W, 1257 m, 25 June 2011, *Johnson & Smith 11-058* (BRY); Trinity County, 10.3 miles north along Hastings Tie Road (FS-4N12), 40.5626°N, 123.5236°W, 1577 m, 27 June 2017, *Johnson & Johnson 17-094* (BRY); Tulare County, Rabbit Meadows vicinity off Forest Service road 14S11 near 14S13A, T14S R29E S18, 2300 m, *Johnson 94-065* (BRY); Tuolumne County, Twaine Harte vicinity, north side of highway 108, just west of hairpin in Lava Drive, 38.0266°N, 120.2369°W, 1159 m, 5 June 2014, *Johnson* et al. *14-077* (BRY); east of the Punch Bowl, north side of 4N12 ca. 5.4 miles east-northeast from junction with highway 108, 38.2386°N, 119.9496°W, 2178 m, 5 June 2014, *Johnson* et al. *14-108* (BRY); **Idaho**: Blaine County, two miles above Alturas Lake, above Alpine Creek, 7400 ft, 23 June 1941, *Cronquist 2661* (IDS); Boise County, at the 62 mile marker of Highway 21, ca. 8 miles south of Lowman, 44.02°N, 115.62°W, 1859 m, 14 July 2003, *Porter & Machen 13772* (BRY); Idaho County, Trail to Lake Serene, 45.1961°N 116.1907°W, 2169 m, 12 July 2013, *Smith* et al. *11162* (BRY, SRP scan); Valley County, FR 626, west of Sagehen Reservoir, Sagehen Basin, 44.3196°N, 116.1547°W, 1845 m, 17 July 2011, *Smith 9922* (BRY, SRP scan); Brundage Mt., 7000 ft, 23 July 1940, *Davis 2930* (IDS); **Nevada**: Douglas County, Carson Range, Genoa Peak Road, 2560 meters, 13 August 1974, *Williams 74-D-83* (RENO scan); Nevada, Washoe County, Carson Range, Little Valley, near bridge, 1981 meters, 22 June 1974, *Tiehm s.n.* (RENO scan); **Oregon**: Crook County, Little Hay Creek vicinity, along Forest Service Road 150 0.1 miles north of jct with forest service road 2610, T13S R19E S22, *Johnson 97-133* (BRY); Harney County, Steens Mountain Loop Byway, 1 km below jct with Kiger Gorge turnoff, 42.69°N, 118.59°W, 8251 ft, 21 July 2007, *Johanson 07-76* (WTU scan); Jackson County, Just north of Hwy 66 along Moon Prairie Rd, 42.1263°N, 122.3321°W, 1161 m, 29 June 2017, *Johnson & Johnson 17-147* (BRY); Josephine County, Upper Biglow Lake, 10 July 1949, *Baker & Ruhle 385* (IDS scan, WTU scan); **Washington**: Asotin County, Anatone Butte vicinity ca. 7.2 miles west of highway 129 on West Mountain Road, 1400 m, 2 July 1994, *Johnson & Johnson 94-049* (BRY); ca. 0.5 mile west of Anatone Butte, 10 miles southwest of Anatone, T7N R45E, S2, 4700 ft, 12 June 1949, *Cronquist 5847* (ID scan, WTU scan WS); Chelan County, Rainbow Creek drainage, ca. 4.5 miles north of Stehekin River, 3000 ft, 19 June 1971, *Naas & Naas 851* (WWB scan); Columbia County, Godman Spring campground, 18.5 miles southwest of Dayton, 46.10°N, 117.7861°W, 1753 m, 12 July 2004, *Legler 2014* (WTU scan); Klickitat County, dry hills, Falcon Valley, 18 July 1908, *Suksdorf 168* (WTU ×3 scan).

#### Notes.

By elevating Navarretia divaricata subsp. vividior to species status, as done below, N. divaricata subsp. divaricata henceforth is designated simply *N. divaricata*. Fresh *N. divaricata* have no noticeable odor. The reported presence of this species in Montana is based on a single, misidentified specimen collected in an agricultural field; that specimen differs from *N. divaricata* in branching structure, having pinnately divided leaves, and possessing different bract and calyx morphology (our determination is *N. squarrosa* (Eschsch.) Hook. & Arn. [MONT-68910 scan!]. Though the protologue provides no mention of *N. divaricata*, Peck’s rationale for recognizing N. prolifera var. breviflora as distinct from *N. divaricata* can be inferred from the key and species descriptions in the first edition of his Manual of the Higher Plants of Oregon (Peck 1941). Examination of the type reveals it is simply a narrow, more erect *N. divaricata* that fits well within the range of variation observed in this species. Peck, perhaps influenced by Mason (1951), came to realize this and removed N. prolifera var. breviflora from the second edition of his manual (Peck 1961).

**Figure 1.**
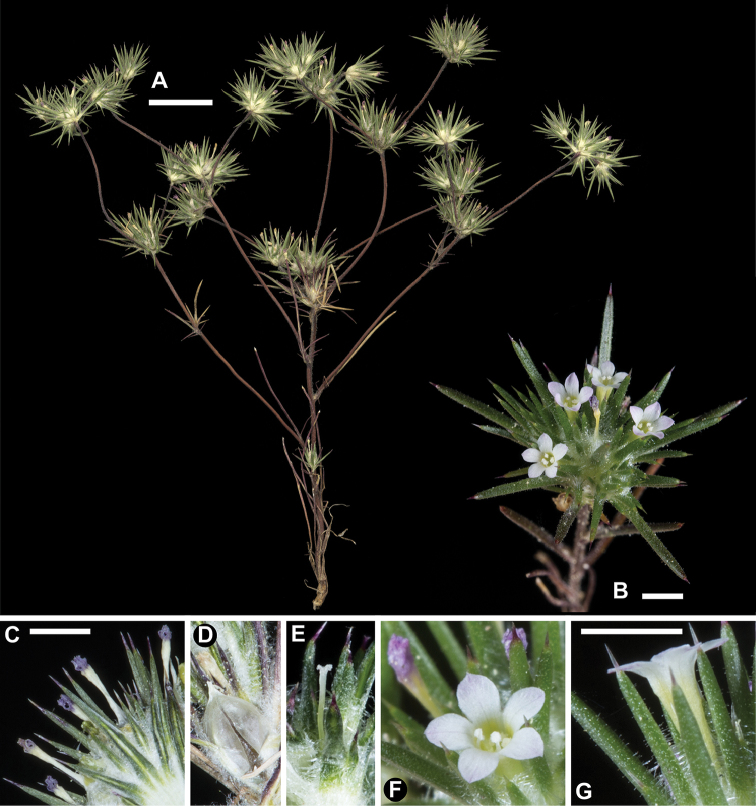
*Navarretia divaricata*. **A** Pressed specimen showing plant habit, scale bar = 1 cm (*Johnson 14-143*) **B** Flowering head in the field, scale bar = 2 mm (*Johnson 15-045*) **C–E** Equivalent magnification, scale bar = 2mm **C** Pressed flowering head showing typical coloration of dried flowers (*Johnson 16-024*) **D** Dried fruit, showing dehiscence from base upward typical in all of the species detailed herein (*Johnson 16-024*) **E** Fresh flowering head dissected with corolla removed to show style and two-lobed stigma (with third lobe nearly entirely fused to one of the two apparent lobes; Johnson 16-024) **F, G** Fresh flowers showing typical coloration (Johnson 15-045), equivalent magnification, scale bar = 2 mm. All photographs by L. A. Johnson and vouchers deposited at BRY.

*Navarretia divaricata* differs from the remaining divaricately branched taxa of *Navarretia* (i.e., those treated below plus *N. crystallina* L.A. Johnson & D.Gowen, *N. miwukensis* D.Gowen & L.A.Johnson, and *N. prolifera*, in having two of its three stigmatic lobes nearly entirely fused, and in having unequal fruit valves with two of the three valves normal sized and bearing a septum, while the third valve is smaller and lacks a septum entirely or nearly so (Cronquist et al. 1984; personal observation). These other species have three lobed stigmas and three equal valves, each bearing a septum. *Navarretia divaricata’s* flowers, ± 4 mm long, are among the smallest in *Navarretia* and typically dry with pink lobes with a white or yellowish throat and tube, though the throat and tube may at times be streaked with red. On herbarium specimens, the contrast between darker, pink lobes and light, whitish or yellowish tube is preserved on many flowers even on specimens 150 years old (Fig. 1). This coloration pattern enables this species to be determined without dissecting flowers to observe the stigma or fruit valves.

**Figure 2.**
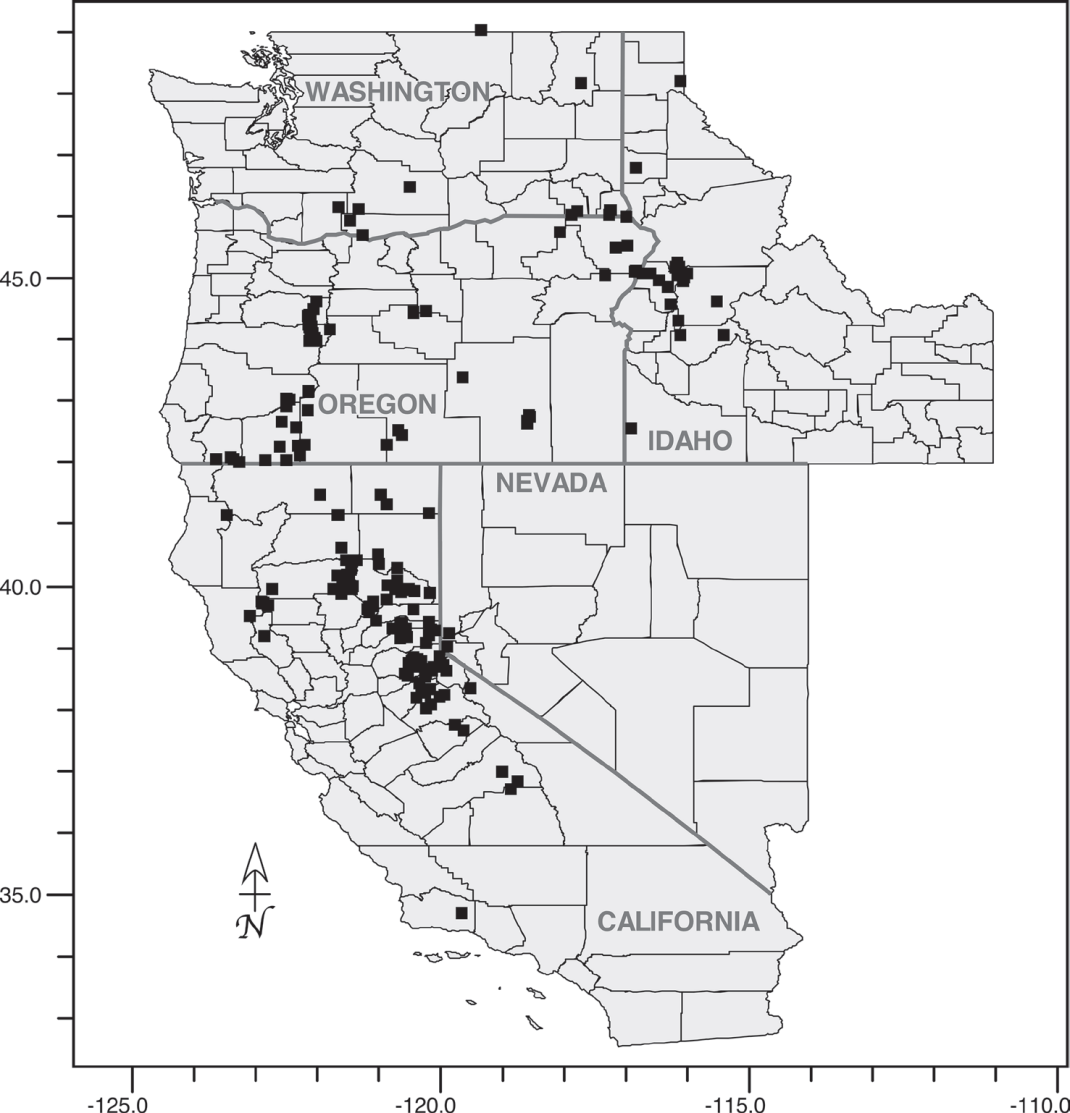
Distribution of *Navarretia divaricata* (black squares) occurrences across its native range in the western United States.

### Navarretia vividior

Taxon classificationPlantaeEricalesPolemoniaceae

(Jeps. & V.L.Bailey) L.A.Johnson & D.Gowen, comb. et stat. nov.

urn:lsid:ipni.org:names:60475570-2

Figs 3 , 4

Navarretia divaricata var. vividior Jeps. & V.L.Bailey, Fl. Calif. 3(2): 156. 1943. Navarretia divaricata subsp. vividior (Jeps. & V.L.Bailey) H.Mason, Ill. Fl. Pacific States 3: 449. 1951.
Type: United States of America. California: Lake County, Mt. Hanna, 15 July 1897, *Jepson 15045* (Holotype: JEPS! [JEPS-2659]). Basionym.

#### Type.

Based on Navarretia divaricata var. vividior Jeps. & V.L.Bailey.

#### Emended description.

***Taprooted annual herbs*** to 12(–15) cm tall and 20(–25) cm wide, sometimes larger, often wider than tall. Primary stem erect, terminating in an inflorescence head 1–2(–4) cm above the cotyledons; generally greatly exceeded by secondary stems, with tertiary, and quaternary stems present on larger plants; higher order branches arise from axils of proximal inflorescence bracts, axils of leaves subtending the primary head, or less commonly, leaves within 1 cm of an inflorescence head; branches ascending to spreading and ± leafless, except for leaves subtending higher order branches or within 1 cm of a head; stem and branches reddish-brown, commonly glandular-villous, the trichomes generally greater than 0.5 mm (often ± 1mm) long, sometimes glabrescent; distal-most branches generally 0.3–0.5 mm in diameter. Cotyledons two, linear, entire, united at base. ***Leaves*** somewhat glandular-pubescent proximally, less so distally; leaves at the lowermost nodes opposite, linear-filiform, and widened at the point of stem attachment, the proximal nodes often congested with overlapping leaf bases. More distal leaves alternate, entire, or more commonly with 1–3(–5) paired or unpaired linear lateral lobes 1–10 mm long attached along the proximal 3–5(–15) mm of the leaf, with an elongated, linear terminal segment. ***Inflorescences*** head-like, largest generally ≥ 12 mm diameter (exclusive of bract tips; ≥ 18 mm with bract tips), mostly 10–25-flowered, glandular-pubescent with some minutely-glandular villous trichomes proximally. Inflorescence bracts < 15(–20) mm long, palmatifid to subpalmatifid; outermost 1–2 bracts with a short achlorophyllous base and 2–3(–4) pairs of lateral lobes flanking an elongate terminal lobe, the distal pair of lateral lobes sometimes shorter and reflexed somewhat out of plane relative to the other lobes; bract bases become larger and clasping centripetally with lateral lobes reduced to a single pair departing from near the apex of the bract base flanking the central terminal lobe, all bract lobes chlorophyllous, entire, long tapering acute. Bracts somewhat minutely-glandular villous abaxially, often more densely villous adaxially and proximally along the lobes just above the bract base, glands becoming more prominent and their stipe diminishing in length toward the bract tips. ***Flowers*** actinomorphic, ***calyces*** mostly 4–7.5(–8.5) mm long, tube ~ 1.5–2.5 mm; costae entire, long tapering acute, strongly unequal to subequal with typically two costae longer than the other three; costae narrowing proximally, the shorter ones narrower at base than the intercostal membrane and the longer ones subequal with the membrane; calyx tube achlorophyllous, glandular-puberulent on the intercostal membrane with the costae at least somewhat glandular-villous, the gland stipes longest along the costae at the junction with the intercostal membrane, diminishing in length toward the chlorophyllous costae tips (may be glabrescent with age); intercostal membrane v-shaped at sinus. ***Corolla*** generally equal or shorter than longest calyx costae at anthesis but exceeding the calyx as fruit matures, narrowly funnelform, glabrous, 5.0–7.2 mm long, lobes 0.8–1.1(–1.4) mm long × 0.6–0.9(–1.2) mm wide, tube white proximally, distal tube white or yellow, throat bluish, sometimes streaked with magenta, lobes medium bluish-lavender; tube base expanding and investing the fruit apex. ***Stamen*** filaments unequal, 0.3–1.2 mm long, inserted unequally 0.3–1.3 mm below corolla sinuses, anthers ± included in throat to exserted less than half the length of the corolla lobes; pollen blue (white rarely?), apertures pantoporate, acolpate; sexine seimitectate, reticulate, heterobrochate. ***Ovary*** three-chambered, stigmatic lobes three, included in to slightly exserted from corolla throat; capsule ~ 2.3–3.3 mm long, dehiscing circumcisally around the base with valves splitting upward. ***Seeds*** generally 5–7(8) per locule, medium brown, ovoid-angular, mucilaginous when wet. ***Nuclear gene*** loci showing allotetraploid PCR amplification patterns.

#### Habitat, distribution, and phenology.

*Navarretia vividior* occurs in soils influenced by volcanic activity with favorable water status such as the edges of ephemeral pools and transient rivulets, to open flats or gentle slopes in forested areas from 300–1600 meters elevation. It occurs predominately in the North Coast Range from Lake and Sonoma Counties, California in the south to Humboldt, Trinity, and the western edge of Shasta County in the north (Fig. 4). A few populations cross the central valley and occur on the western flank of the northern Sierra Nevada/southern Cascade Range in Butte County, California, where they flower in late May–early June, rather than late June–July as is typical for species in the North Coast Range.

#### Conservation status.

As defined here, *Navarretia vividior* is distributed more narrowly than previously considered. Some historical populations have not been revisited for ±100 years, though other known occurrences have been revisited multiple times over the past 20 years with no apparent change in local numbers (beyond what may be expected during drought years). Following IUCN (2012) Red List version 3.1 criteria, this species is most accurately characterized as data deficient, though it likely borders between being Vulnerable to a species of Least Concern.

#### Etymology.

This specific epithet is derived from the Latin *vividus*, lively or vigorous, with the comparative ending -*ior*, more so, in reference to the larger, more robust habit and flowering heads this taxon possesses in comparison to *N. divaricata*. Alternative epithets of var. vividia and var. fertilior were considered by Jepson and Bailey for this entity, as indicated by annotations on the type sheet.

#### Representative specimens examined.

**UNITED STATES OF AMERICA. California**: Butte County, North of Magalia at Old Coutelenc Site, along Coutelenc Rd, 1.7 miles south of junction with Skyway at Lovelock, 17 June 1980, *Schlising & Banchero 3739* (CHSC); Upper Bidwell Park, Chico, on the north rim overlooking Sycamore Creek Canyon about 1.8 miles east of the power lines, 1280 ft, 30 May 1983, *Oswald 578* (CHSC); South of Cohasset off of Cohasset Highway, T23N R2W S8, 4 June 1979, *Schlising 3435* (CHSC); Colusa County, Along Pacific Ridge Road (16N06) 0.5 miles east of road to Spanish Ridge and Hough Springs, 39.2236°N, 122.625°W, 3450 ft, 3 July 1998, *Janeway & Isle 5711* (CHSC); Humboldt County, Waif on river bar, valley of Van Duzen River opposite Buck Mtn, 1000 ft, 27 June–30 July 1908, *Tracy 2721* (UC); Little Van Duzen River, 23 June 1937, *Eastwood & Howell 4813* (CAS); Lake County, Mt. St. Helena, 6 June 1915, *Eastwood 4706*; Bartlett Springs, 9 June 1938, *Jepson 18938* (JEPS); Boggs Lake, at base on Mount Hanna, 29 June 1945, *Mason 12629* (DS); Margin of Boggs Lake, 3000 ft, 29 June 1945, *Baker 11088* (CAS); 3.5 miles by road east of Seigler Springs, margin of Snow’s Lake, 2150 ft, 26 June 1956, *Crampton 3646* (AHUC); About 1/4 mile down Harrington Flat Road from Bottle Rock Rd, where access road follows under powerlines, 2850 ft, 7 June 1981, *Knight & Knight 4148* (CAS); Bogg’s Lake Vicinity, between Harrington Flat Road and the Bogg’s Lake parking area, to the north side of road along the power line corridor, 38.8904°N, 122.7837°W, 862 m, 13 May 2004, *Johnson 04-032* (BRY), and 12 July 2016, *Johnson 16-029* (BRY); Bartlett Mountain Ranger Station, 24 Sept 1949, *Crampton 494B* (AHUC, DAV); About 6.3 miles from Hwy 20 along Bartlett Spring Road, at junction with Pinnacle Rock Road, 39.1345°N, 122.7769°W, 3915 ft, 10 May 2005, *Johnson 05-021* (BRY) and 12 July 2016, *Johnson 16-032* (BRY); Mendocino County, Chamise Mt. north of Bell Springs, 3500 ft, 9 July 1934, *Tracy 13326* (UC); Jeep trail above Bell Springs Rd 11 miles south of jct with Harris Rd, 39.9667°N, 123.6113°W, 1092 m, 28 June 2017, *Johnson & Johnson 17-119* (BRY); Eden Valley Ranch, vernal pool area, north of Hearst, 1249.6 m, 11 July 1978, *Wheeler 506* (CAS); Napa County, Gravelly slope in chaparral, edge of the Crater Country, 13 July 1930, *Howell 5347* (CAS); Robert Louis Stevenson State Park; along trail to Table Rock on East side of Hwy from parking area off Hwy 29, 38.6513°N, 122.5867°W, 802 m, 30 May 2009, *Johnson & Gowen 09-049* (BRY); Shasta County, Harrison Gulch Rd, 2.4 miles north of Hwy 36 west of Platina, T29N R10W S3, 3100 ft, 1 July 1996, *Oswald, Ahart & Ondricek-Fallsheer 7906* (CHSC) and 13 July 2016, *Johnson 16-062* (BRY); 0.3 miles north of Hwy 36 along NF-29, 40.3699°N, 122.9380°W, 893 m, 27 June 2017, *Johnson & Johnson 17-052* (BRY); Sonoma County, Mayacmas Mtns, Caldwell Pines, ca. 3.5 miles west of Glenbrook and Bottle Rock Rd, 38.841°N, 122.815°W, 2350–3000 ft, 27 June 1975, *Neilson & Sholars 3294b* (DAV); Tehama County, West of Red Bluff and south of Platina on Forest Route 45 (Tedoc Mtn. Rd), 6.3 miles south of Hwy 36, 3800 ft, 12 July 1995, *Oswald & Ahart 7022* (CHSC) and 13 July 2016, *Johnson 16-058* (BRY); Trinity County, Along Wildwood Rd north of Hwy 36 between mile post 7 and 8, where drainage crosses and goes under road, 24 June 2005, *Gowen 452* (BRY, JEPS) and 13 July 2016, *Johnson 16-063b* (BRY); 6.2 miles north of Hwy 36 along Hastings Tie Road, 40.5092°N, 123.5041°W, 1597 m, 27 June 2017, *Johnson & Johnson 17-082* (BRY).

#### Notes.

*Navarretia vividior* are mephic when fresh. *Crampton 494B* [AHUC-38375] (collected by McMillan at the Bartlett Mountain Ranger Station) is a voucher for a tetraploid chromosome count from pollen mother cells. Our low copy nuclear gene data indicate this species is an allotetraploid, with *N. modocensis* the paternal diploid parent and *N. aeroides* the maternal diploid parent. Four of the eight paratypes listed for N. divaricata var. vividior belong here: *Chestnut & Drew s.n*. [UC-52826], *Tracy 2721* [UC-133661], *Tracy 13326* [JEPS-70300], and *Jepson 18938* [JEPS-70299]; a fifth paratype, *Condit s.n.* [UC-455660], is difficult to place with confidence; it was collected from ‘garden soil’ and thus its original source is uncertain, but its smaller corolla features suggest *N. vividior*. The *Torrey 302* syntype of *Gilia divaricata* Torr. ex Gray also belongs here.

**Figure 3.**
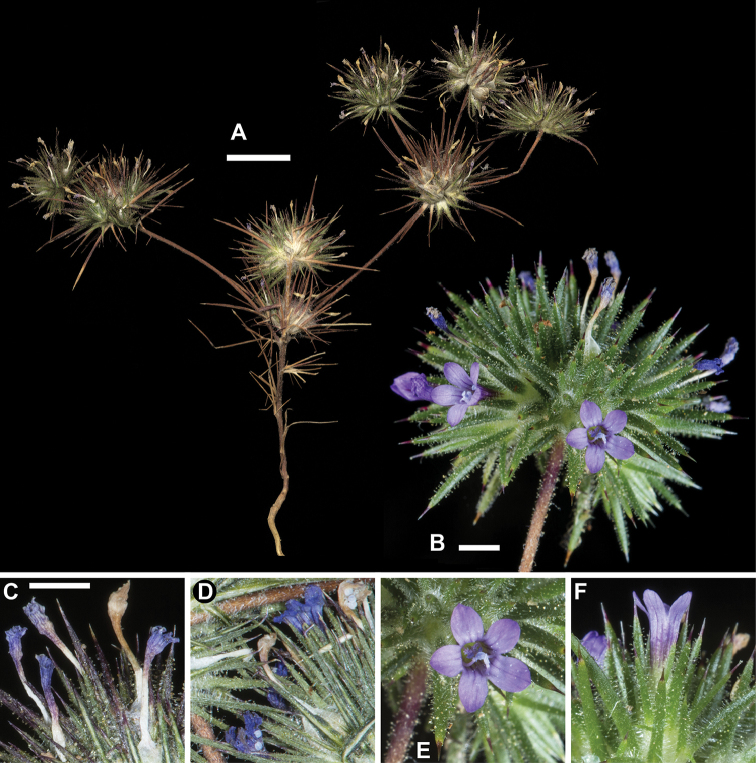
*Navarretia vividior*. **A** Pressed specimen showing plant habit, scale bar = 1 cm (*Johnson 16-066*) **B** Flowering head in the field, scale bar = 2 mm (*Johnson 16-063b*) **C–F** Equivalent magnification, scale bar = 2mm **C, D** Pressed flowering head showing typical coloration of dried flowers and blue pollen (*Johnson 16-029, 16-063b*, respectively) **E, F** Fresh flowers showing typical coloration (*Johnson 16-063b*). All photographs by L. A. Johnson and vouchers deposited at BRY.

*Navarretia vividior* is readily distinguished from *N. divaricata* with its larger flowering head, larger flowers, equally divided 3-lobed stigma, typically blue pollen, and bluish-lavender corolla lobes and throat (Fig. 3). Stems are typically larger in diameter and invested with long, gland tipped hairs. The evenly 3-lobed stigmas, larger flowers, and some variation in flower color with colored lobes, throat, or both apply to three additional species that have heretofore generally fallen under the umbrella of N. divaricata subsp. vividior. These species are described and differentiated below.

**Figure 4.**
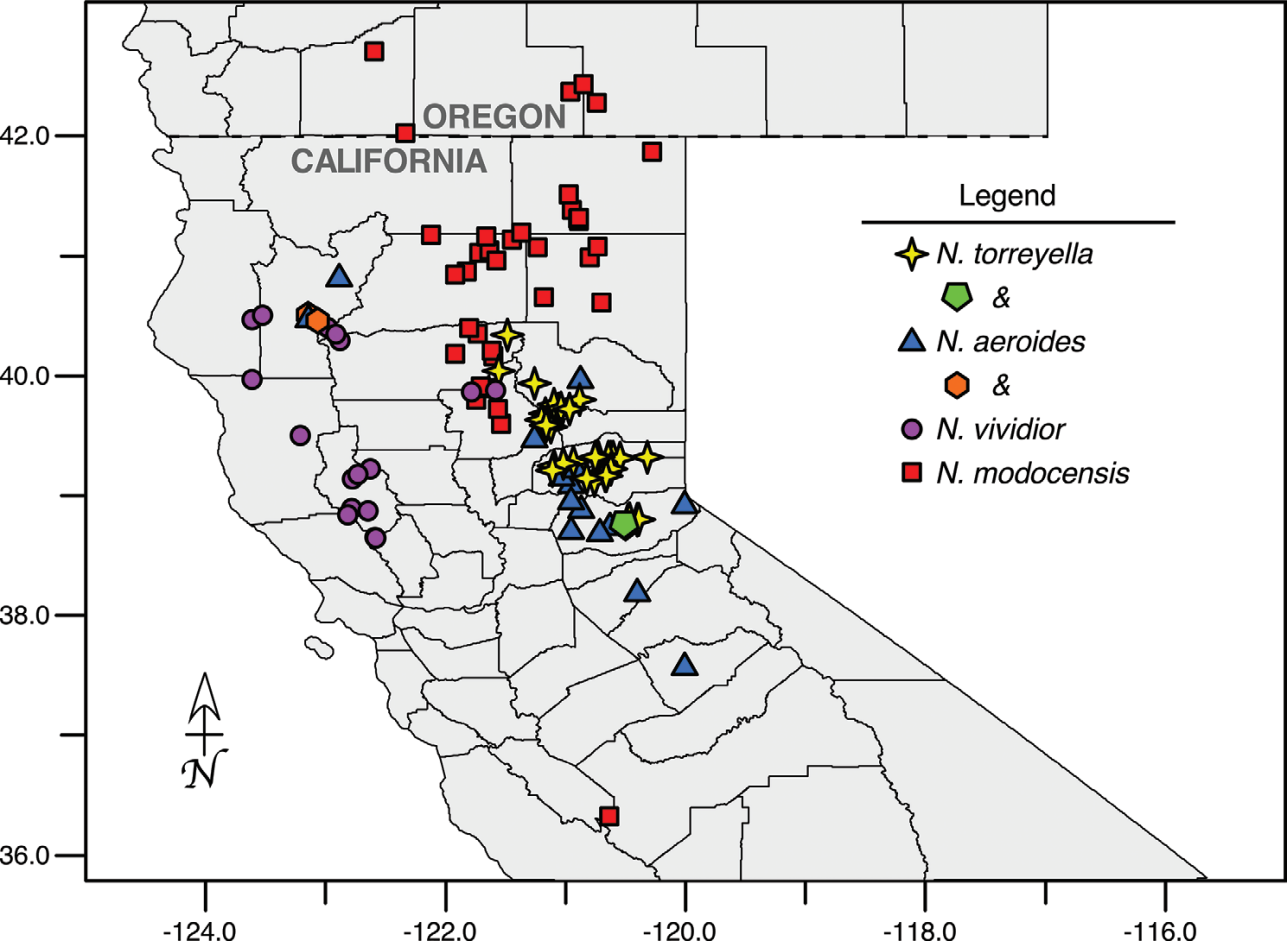
Distribution of *Navarretia vividior* (circles), *N. modocensis* (squares), *N. aeroides* (triangles), and *N. torreyella* (4-pointed stars) occurrences across their native range in California and southern Oregon, United States. Instances of observed syntopy are indicated with the hexagon and pentagon, as defined in the legend.

### Navarretia modocensis

Taxon classificationPlantaeEricalesPolemoniaceae

L.A.Johnson & D.Gowen sp. nov.

urn:lsid:ipni.org:names:60475567-2

Figs 4 , 5

#### Type.

United States of America. California: Shasta County, west side of Hwy 89, ca 10 miles north of junction with Hwy 299. Meadow area adjacent to railroad tracks, 41.0450°N, 121.6326°W, 2992 ft, 17 June 2004, *L.A. Johnson 04-130* (holotype BRY! [BRY-614952]; isotypes JEPS! RSA! and to be distributed).

#### Diagnosis.

A species similar to *Navarretia vividior*, but distinguished by being diploid, rather than allotetraploid, generally being more conspicuously villous in the proximal inflorescence, possessing slightly larger flowers and more exserted corollas that tend toward pinkish-lavender lobes with darker throat above a yellowish tube rather than bluish to bluish-lavender lobes with darker throat above a yellowish or whitish tube, and having white (rarely blue) rather than blue pollen.

#### Description.

***Taprooted annual herbs*** to 12(–15) cm tall and 20(–25) cm wide, sometimes larger, often wider than tall. Primary stem erect, terminating in an inflorescence head 1–2(–4) cm above the cotyledons; generally greatly exceeded by secondary stems, with tertiary, and quaternary stems present on larger plants; higher order branches arise from axils of proximal inflorescence bracts, axils of leaves subtending the primary head, or less commonly, leaves within 1 cm of an inflorescence head; branches ascending to spreading and ± leafless, except for leaves subtending higher order branches or within 1 cm of a head; stem and branches reddish-brown, commonly glandular-villous, the trichomes generally greater than 0.5 mm (often ± 1mm) long, sometimes glabrescent; distal-most branches generally 0.3–0.5 mm in diameter. Cotyledons two, linear, entire, united at base. ***Leaves*** somewhat glandular-pubescent proximally, less so distally; leaves at the lowermost nodes opposite, linear-filiform, and widened at the point of stem attachment, the proximal nodes often congested with overlapping leaf bases. More distal leaves alternate, entire, or more commonly with 1–3(–5) paired or unpaired linear lateral lobes 1–10 mm long attached along the proximal 3–5(–15) mm of the leaf, with an elongated, linear terminal segment. ***Inflorescences*** head-like, largest generally ≥ 12 mm diameter (exclusive of bract tips; ≥ 18 mm with bract tips), mostly 12–20-flowered, sometimes more, glandular-pubescent, generally with conspicuous, minutely-glandular villous trichomes proximally. Inflorescence bracts < 15(–20) mm long, ± palmatifid to subpalmatifid; outermost 1–2 bracts with a short achlorophyllous base and 2–3 pairs of lateral lobes flanking an elongate terminal lobe, the distal pair of lateral lobes sometimes shorter and reflexed somewhat out of plane relative to the other lobes; bract bases become larger and clasping centripetally with lateral lobes reduced to a single pair departing from near the apex of the bract base flanking the central terminal lobe, all bract lobes chlorophyllous, entire, long tapering acute. Bracts minutely-glandular villous abaxially, often more densely villous adaxially and proximally along the lobes just above the bract base, glands becoming more prominent and their stipe diminishing in length toward the bract tips. ***Flowers*** actinomorphic, ***calyces*** mostly 5–7.5(–12) mm long, tube ~ (1.5–)2–2.5(–3) mm; costae entire, long tapering acute, strongly unequal to subequal with typically two costae longer than the other three; costae narrowing proximally, the shorter ones narrower at base than the intercostal membrane and the longer ones subequal with the membrane; calyx tube achlorophyllous, glandular-puberulent on the intercostal membrane with the costae at least somewhat glandular-villous, the gland stipes longest along the costae at the junction with the intercostal membrane, diminishing in length toward the chlorophyllous costae tips (may be glabrescent with age); intercostal membrane v-shaped at sinus. ***Corolla*** generally equal to exceeding the calyx costae at anthesis and exceeding the calyx further as fruit matures, narrowly funnelform, glabrous, 6.0–8.2 mm long, lobes (1.0–)1.2–1.5(–1.95) mm long × 0.8–1.4 mm wide, tube white proximally, yellow distally, throat lavender–purple or purplish streaked, lobes light to dark pinkish-lavender; tube base expanding and investing the fruit apex. ***Stamen*** filaments unequal, 0.3–1.3 mm long, inserted unequally 0.3–1.6 mm below corolla sinuses, anthers ± included in throat to exserted less than half the length of the corolla lobes; pollen white (uncommonly blue), apertures pantoporate, acolpate; sexine seimitectate, reticulate, heterobrochate. ***Ovary*** three-chambered, stigmatic lobes three, included in to slightly exserted from corolla throat; capsule ~ 2.6–3.8 mm long, dehiscing circumcisally around the base with valves splitting upward. ***Seeds*** generally 4–9 per locule, medium brown, ovoid-angular, mucilaginous when wet. ***Nuclear gene*** loci showing diploid PCR amplification patterns.

**Figure 5.**
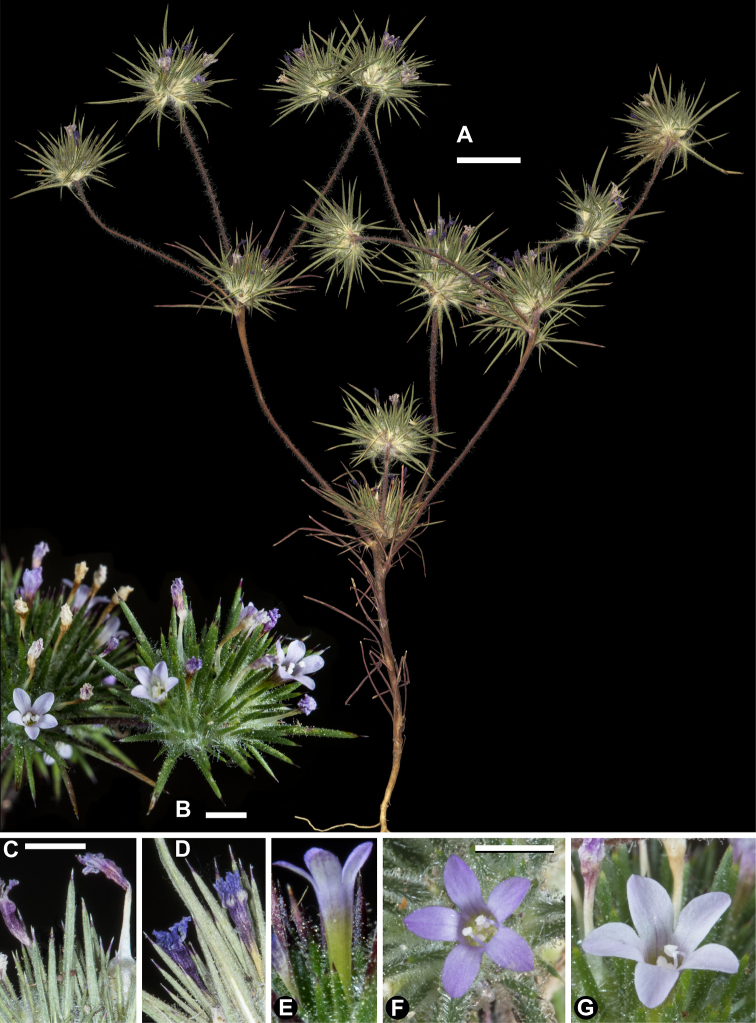
*Navarretia modocensis*. **A** Pressed specimen showing plant habit, scale bar = 1 cm (*Johnson 04-130*) **B** Flowering head in the field, scale bar = 2 mm (*Johnson 16-072*) **C, D** Pressed flowering heads showing range of typical coloration, equivalent magnification, scale bar = 2 mm (*Johnson 04-124, 04-130*, respectively) **E–G** Fresh flowers showing typical coloration and white pollen, equivalent magnification, scale bar = 2 mm. (**E, F** = *Johnson 14-170*; **G** = *Johnson 16-0*72). All photographs by L. A. Johnson and vouchers deposited at BRY.

#### Habitat, distribution, and phenology.

*Navarretia modocensis* occurs in volcanic influenced soils in forest openings and sagebrush slopes from (390–)800–1700 meters predominately in the Modoc Plateau of northeastern California and adjacent southern Oregon, but extending south to the western flank of the northern Sierra Nevada/southern Cascade Range in Butte County, California, and with a long-distance disjunct occurrence in San Benito County, California. Flowering occurs primarily in (April–)June–July.

#### Conservation status.

*Navarretia modocensis* has many occurrences throughout its range and is often abundant when encountered. It is a species of Least Concern following IUCN (2012) Red List version 3.1 criteria.

#### Etymology.

From the Latin -*ensis*, origin or place, combined with Modoc, in reference to the Modoc Plateau on which this taxon predominantly (but not exclusively) occurs.

#### Representative specimens examined

**(paratypes)**. **UNITED STATES OF AMERICA. California**: Butte County, North east of Forest Ranch, 2.3 miles east and southeast of Hwy 32 along Garland Road, T24N R3E S33, 2960 ft, 13 June 1980, *Schlising & Azevedo 3699* (CHSC) and 20 May 2017, *Johnson 17-027* (BRY); Top of North Table Mt, ca 200 meters north of junction of Cherokee Rd and east edge of Mt, T20N R4E S17 NE1/4, 18 May 1979, *Jokerst, Devine, & Greenstein 0462* (CHSC); Ponderosa way on Musty Buck Ridge between Cohasset and Big Chico Creek, T24N R2E S25 SE NW 1/4, 2500 ft, 16 May 1989, *Oswald 3773* (CHSC); About 0.5 mile east of Kunkle Creek, about 2.5 miles south-east of Paradise, 1500 ft, 29 May 1979, *Ahart 1906* (CHSC); Lassen County, Off Hwy 139, up slope and across fence at a pull out area on west side of road, near mile marker 49.5, 40.9833°N, 120.7950°W, 1615 m, 9 July 2014, *Johnson 14-176* (BRY); Murrer Meadow Rd, ca 3 miles east of Eagle Lake Biological Station, 5100 ft, 24 June 1967, *Stern 2418* (CHSC); 100 yards south of Hwy 44, 34 miles northwest of Susanville, 26 June 1974, *Stern 5863* (CHSC); Ash Valley RNA/ACEC, T37N R11E S5, 5100 ft, 29 June 1993, *Schoolcraft 2303* (CAS; mixed collection with *N. divaricata*); Near Bar Springs road, just off Hwy 299 near top of grade, ca. 7.4 miles east of county line, 41.0669°N, 121.2315°W, 1383 m, 14 July 2016, *Johnson 16-075* (BRY); Modoc County, Vicinity of mile maker 9.37 off of Hwy 299, near Roney Flat and Johnson Creek Roads, 41.30602°N, 120.89584°W, 1428 m, 25 June 2011, *Johnson & Smith 11-064* (BRY); Fandango Pass vicinity, helicopter pad 5.1 miles from Hwy 395 on Fandango Pass Road (Rd 9) at south end of Buck Creek Fire station and intersection with Jack’s Rd, 41.8639°N, 120.2849°W, 1586 m, 9 July 2014, *Johnson 14-170* (BRY); About 0.3 miles up 41N25 (Shake Canyon) off of Hwy 139/299, 41.3825°N, 120.9437°W, 1415 m, 9 July 2014, *Johnson 14-173* (BRY); 0.6 miles along Johnson Creek road from jct with Hwy 299/139 about 9.5 miles north of county line, 41.3120°N, 120.8874°W, 1479 m, 14 July 2016, *Johnson 16-083* (BRY); 0.7 miles east of Hwy 139 along road FS 46, 41.5090°N, 120.9750°W, 1519 m, 14 July 2016, *Johnson 16-086* (BRY); Little Hot Spring Valley, 15 June 1894, *Baker s.n.* (UC 23519); San Benito County, Condon Peak vicinity, 36.3205°N, 120.63817°W, 4683 ft, 5 June 2016, *O’Dell s.n*. (BRY; JEPS); Shasta County, Fuller Flat. north side of Hwy 299, just west of mile marker 64.71, about 14 miles west of junction with Hwy 89, 40.86532°N, 121.8238°W, 3792 ft, 17 June 2004, *Johnson 04-124* (BRY, JEPS, RSA) and 14 July 2016, *Johnson 16-072* (BRY); Meadow 0.3 miles past Rock Creek, south side of road 37, 41.0245°N, 121.7164°W, 3180 ft, 17 June 2004, *Johnson 04-137* (BRY); Rock Creek vicinity; north of Summit Lake Road (Forest Route 38N10) ca. 3.5 miles from junction with Clark Creek Road, east of Lake Britton & Hwy 89, 41.0218°N, 121.7217°W, 973 m, 17 June 2005, *Johnson & Zhang 05-157* (BRY); 0.4 miles south of Hwy 299 along Cassel road, 40.9561°N, 121.5772°W, 926 m, 14 July 2016, *Johnson 16-073*; Ahjumawi Lava Springs State Park, 3 miles north of McArthur, along Spatter Cone Trail, 3450 ft, 24 May 2004, *Fischer & Marr 0213* (CHSC); Fall River Springs, 3500 ft, June 1903, *Hall & Babcock 4213* (UC); Montgomery Creek, 27 June 1912, *Eastwood 631* (CAS); Willow Creek Ranch about 6.8 miles south of the intersection of California State Rd 89 and Squaw Valley Rd in McCloud, 2900 ft, 29 May 1995, *Almeda & Eisenhardt 7417* (CAS); Tehama County, High Cascade Range. Along Road 27N08 to Deer Creek and Onion Butte, ca. 4.6 miles from jct with Hwy 32 at sharp hairpin bend (ca. 0.9 miles from Deer Creek), 40.1624°N, 121.5999°W, 994 m, 11 June 2015, *Johnson 15-047* (BRY); North side of Hwy 36 in ecotone gradation between mixed conifer forest and chaparral at mile marker 75.49, about 12.8 miles west of jct with Hwy 89 to Mt. Lassen, 40.34734°N, 121.7299°W, 1138 m, 12 June 2015, *Johnson 15-057* (BRY); Along Ponderosa Way at crossing of Soap Creek; , 40.3933°N, 121.8033°W, 2450 ft, 30 May 1997, *Taylor 16013* (BRY, JEPS); North side of Ishi Wilderness at Rancheria Creek Trailhead, 40.2052°N, 121.6141°W, 3210 ft, 31 May 2003, *Hillaire & Elliott 327* (CHSC); 33 meters above Road 27N08, 2.3 km east of major hairpin turn, 40.1586°N, 121.6019°W, 1000 m, 30 May 2004, *Janeway & Castro 8123* (CHSC); North facing slope along Powerline Road just southwest of north fork of Little Antelope Creek, 40.1833°N, 121.9186°W, 1890 ft, 25 April 2009, *Castro & Janeway 1778* (CHSC); **Oregon**: Jackson County, Near trail, 19 June 1931, *Howell 6766* (CAS); 7 miles southwest of Prospect, 2100 ft, 28 June 1939, *Hitchcock and Martin 4992* (IDS; OSC); On Copco Rd., about 1/2 mile south of Ranch, just north of California line, T41S R4E S10 SW1/4, 18 June 1990, *Rolle 237* (OSC); Klamath County, 11 miles east of Blye, 30 June 1937, *Peck 19655* (WILLU); Hwy 140, about 4.7 miles west of Lake County line, to south of highway near old dirt road, 12 July 1995, *Johnson 95-053* (BRY); Lake County, 24 miles northwest of Lakeview, 25 June 1927, *Peck 15226* (WILLU); jct of FS-3870 and highway 140, 42.2632°N, 120.7197°W, 1529 m, 29 June 2017, *Johnson & Johnson 17-159* (BRY).

#### Notes.

*Navarretia modocensis* are mephic when fresh. Three of the nine paratypes listed for N. divaricata var. vividior belong here: *Baker s.n.* [UC-23519], *Hall & Babcock 4213* [UC-127856], and *Austin 827* [UC-133706]. A collection from Yosemite National Park, *Mason 12480a* [UC-908393], likely belongs here also, representing a second disjunct population for this species. Pollen is usually white in *N. modocensis*, but blue pollen has been observed.

*Navarretia modocensis* is the species most likely to be visually confused with *N. vividior*. Though *N. modocensis* can have larger inflorescence heads, calyces, and flowers than *N. vividior*, the range of measurements in these features overlap. We are fairly confident (on fresh flowers) that *N. modocensis* has a yellow corolla tube on fresh flowers, and that populations with blue pollen are uncommon, but we are less certain that *N. vividior* always has yellow on its tube, or that populations with white pollen do not exist (given white results from the absence of pigment; we have collected species in several genera, including *Navarretia* that are characterized by blue pollen but occasionally have populations with white pollen). The more robust features of *N. modocensis* contrast more sharply with *N. aeroides*, which has smaller flowers, smaller inflorescence heads, thinner branches, and is visually much more glandular and less villous in its inflorescence heads. The smaller dimensions and very different corolla coloration patterns also readily distinguish *N. divaricata* and *N. torreyella* from *N. modocensis*.

### Navarretia aeroides

Taxon classificationPlantaeEricalesPolemoniaceae

L.A.Johnson & D.Gowen sp. nov.

urn:lsid:ipni.org:names:60475568-2

Figs 4 , 6

#### Type.

United States of America. California: Calaveras County, west of Avery, at the end of a short forest service road off of Avery Sheep Ranch Road, 38.19488°N, 120.39969°W, 3695 ft,17 June 2015, *D. Gowen, 1303* (holotype BRY! [BRY-627257]; isotypes JEPS! RSA!).

#### Diagnosis.

A species similar to *Navarretia vividior*, but distinguished by being diploid, rather than allotetraploid, and being less robust in all respects; *N. aeroides* has smaller inflorescence heads that are conspicuously stipitate-glandular throughout (sometimes inconspicuously villous proximally), thinner branches, tends toward smaller corollas, and has stem trichomes mostly 0.5mm or less rather than mostly ± 1 mm.

#### Description.

***Taprooted annual herbs*** to 9(–12) cm tall and 15(–22) cm wide, sometimes larger, often wider than tall. Primary stem erect, terminating in an inflorescence head 1–2(–4) cm above the cotyledons; generally greatly exceeded by secondary stems, with tertiary, and quaternary stems present on larger plants; higher order branches arise from axils of proximal inflorescence bracts, axils of leaves subtending the primary head, or less commonly, leaves within 1 cm of an inflorescence head; branches ascending to spreading and ± leafless, except for leaves subtending higher order branches or within 1 cm of a head; stem and branches reddish-brown, glandular-pubescent or sparingly so, the trichomes mostly less than 0.5 mm long; distal-most branches filiform, generally no more than 0.3 mm in diameter. Cotyledons two, linear, entire, united at base. ***Leaves*** somewhat finely stipitate-glandular proximally, less so distally; leaves at the lowermost nodes opposite, linear-filiform, and widened at the point of stem attachment, the proximal nodes often congested with overlapping leaf bases. More distal leaves alternate, entire, or more commonly with 1–3(–5) paired or unpaired linear lateral lobes 1–8 mm long attached along the proximal 3–5(–15) mm of the leaf, with an elongated, linear terminal segment. ***Inflorescences*** head-like, generally ≤ 10 mm diameter (exclusive of bract tips; ~15 mm with bract tips), mostly less than 10-flowered, sometimes more, ± conspicuously glandular. Inflorescence bracts < 10(–12) mm long, ± palmatifid to subpalmatifid; outermost 1–2 bracts with a short achlorophyllous base and 2–3 pairs of lateral lobes flanking an elongate terminal lobe, the distal pair of lateral lobes sometimes shorter and reflexed somewhat out of plane relative to the other lobes; bract bases become larger and clasping centripetally with lateral lobes reduced to a single pair departing from near the apex of the bract base flanking the central terminal lobe, all bract lobes chlorophyllous, entire, long tapering acute. Bracts sparsely glandular-villous abaxially, more densely glandular-villous adaxially and proximally along the lobes just above the rachis, with the stipe of each gland diminishing in length toward the bract tips. ***Flowers*** actinomorphic, ***calyces*** mostly 4.0–6.0(–8.5) mm long, tube ~ 1.3–2.2–(2.5) mm; costae entire, long tapering acute, strongly unequal to subequal with typically two costae longer than the other three; costae narrowing proximally, the shorter ones narrower at base than the intercostal membrane and the longer ones subequal with the membrane; calyx tube achlorophyllous, glandular-puberulent on the intercostal membrane and proximal costae, gland stipes lengthen on the costae at the junction with the intercostal membrane, diminishing in length toward the chlorophyllous costae tips; intercostal membrane v-shaped at sinus. ***Corolla*** generally ± equal to the calyx costae at anthesis but exceeding the calyx as fruit matures, narrowly funnelform, glabrous, 4.2–6.0 mm long, lobes 0.75–1.3 mm long × 0.5–0.9(–1.0) mm wide, tube white, transitioning to a bluish throat and lobes in some populations (drying bluish purple, with the distal tube showing hints of magenta or somewhat brownish) or remaining white in others (drying with white or light blue lobes and throat with brownish or magenta distal tube); tube base expanding and investing the fruit apex. ***Stamen*** filaments unequal, 0.2–0.55 mm long, inserted unequally 0.4–0.9 mm below corolla sinuses, anthers included in throat to slightly exserted; pollen blue or white, generally matching corolla lobe coloration, apertures pantoporate, acolpate; sexine seimitectate, reticulate, heterobrochate. ***Ovary*** three-chambered, stigmatic lobes three, included in to slightly exserted from corolla throat; capsule ~ 2.4–3.4 mm long, dehiscing circumcisally around the base with valves splitting upward. ***Seeds*** generally 4–8 per locule, medium brown, ovoid-angular, mucilaginous when wet. ***Nuclear gene*** loci showing diploid PCR amplification patterns.

**Figure 6.**
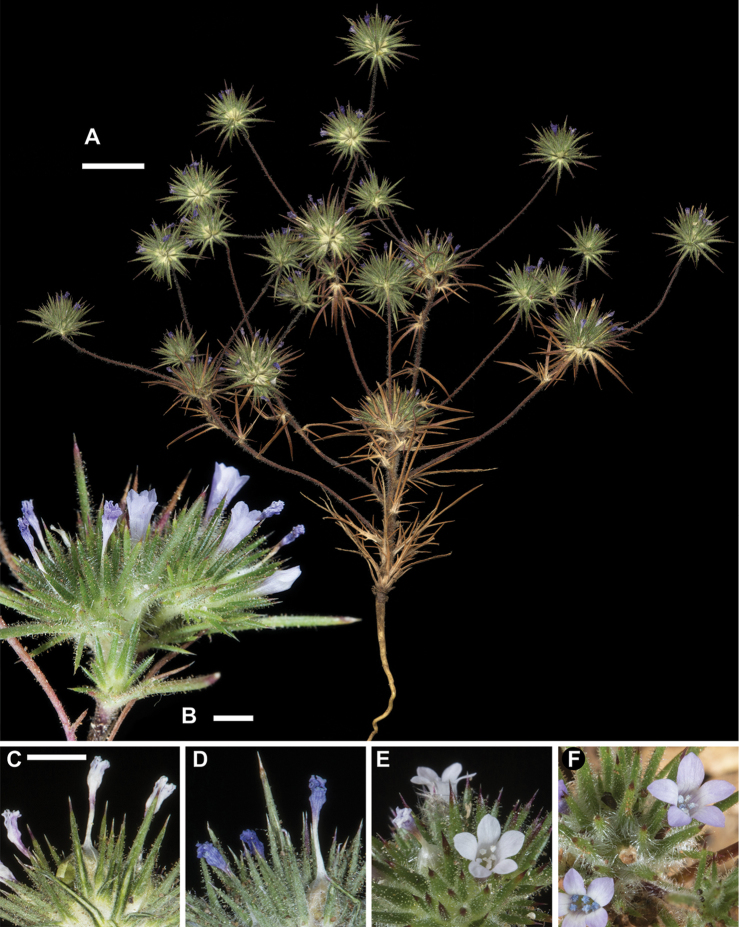
*Navarretia aeroides*. **A** Pressed specimen showing plant habit, scale bar = 1 cm (*Gowen 1303*) **B** Flowering head in the field, scale bar = 2 mm (*Johnson 14-142*) **C–F** Equivalent magnification, scale bar = 2mm **C, D** Pressed flowering head showing range of coloration in dried flowers (*Johnson 16-070, Gowen 1303*, respectively) **E, F** Fresh flowers showing range of coloration (*Johnson 16-063a, Johnson 15-065*, respectively). All photographs by L. A. Johnson and vouchers deposited at BRY.

#### Habitat, distribution, and phenology

*Navarretia aeroides* prefers (reddish) clay soils in forest openings from 400–1350(–1900) meters elevation. Occurrences are widely scattered in the Sierra Nevada from Mariposa County, California in the south to Plumas County in the North, and in the Trinity mountains of the North Coast Range, California. This species flowers primarily June–July.

#### Conservation status.

Many historical collections of this species are sufficiently general in their locality descriptions in areas now populated that our efforts to relocate them, compounded by recent drought years, have been unsuccessful. On the other hand, all but two of our collections were made serendipitously, in the course of looking for other species, suggesting our present knowledge of occurrences is incomplete. Following IUCN (2012) Red List version 3.1 criteria, this species is most accurately characterized as data deficient, though it may be Vulnerable based on the fragmented nature of a limited number of occurrences.

#### Etymology.

From the Latin *aeroides*, like the sky or sky-blue, in reference to the typical color of the corolla.

#### Representative specimens examined

**(paratypes)**. **UNITED STATES OF AMERICA. California**: El Dorado County, Lake Valley, July 1908, *Brandegee s.n.* (UC; mixed collection with *N. divaricata*); 3 miles east of Camino at C.C.C. Camp Snowline, 3400 ft, 6 July 1943, *Robbins 1247* (JEPS, UC); 0.1 mi north of Rescue, along deer valley road, 1300 ft, 30 June 1945, *Robbins 2015* (UC); 3 miles southeast of Greenwood, Coloma Canyon, 18 June 1957, *Crampton 4233* (AHUC); 6.4 miles east of Diamond Springs, 6 June 1960, *Crampton 5546* (AHUC); Sly Park Vicinity. Park Creek Road off of county road E16, 38.7493°N, 120.4970°W, 1207 m, 8 July 2014, *Johnson 14-142* (BRY, JEPS); Mariposa County, Telegraph Hill Road, ca. 7 miles from Hwy 140 via E. Whitelock Road, 37.5773°N, 120.0038°W, 809 m, 9 June 2015, *Johnson 15-005* (BRY, JEPS); Nevada County, Colfax, 3 July 1882, *Jones 3417.* (POM, scan); Colfax, 20–22 Jun 1912, *Eastwood 498* (CAS, UC); Rattlesnake Creek, 5 miles south of Grass Valley, 2000 ft, 29 May 1926, *Mason 3279* (UC); Bed of dried pond west of Greenhorn Creek, T16N R9E S24, 3000 ft, 27 August 1954, *Raven 7980* (CAS); Placer County, Nevada City, Hospital Farm, Sierra Nevada Mtns, 5000 ft, 31 July 1915, *Brainerd & Baird 236* (JEPS); 10 miles west of Forest Hill, 15 June 1955, *Crampton 2921* (AHUC); Plumas County, 2.5 miles northeast of Quincy Junction on Mt Hough-Crystal Lake Road, T24N R10E S4, 4200 ft, 24 June 1981, *Barbe, Fuller, & Howell 3181* (CHSC); East of Quincy, about 2.3 miles along Mt. H road from its junction with Quincy Jct Road, at intersection with 25N14, 39.9747°N, 120.8761°W, 1321 m, 12 June 2015, *Johnson 15-065* (BRY, JEPS) and 14 July 2016, *Johnson 16-087* (BRY, JEPS); Trinity County, Hills south above Hayfork, between Kingsbury Rd. and Bridge Gulch Rd, 25 June 2005, *Gowen 462*; East side of Hwy 3 south of Clair Engle Lake, ca 2.3 miles south of Tannery Gulch Road, 40.8225°N, 122.8887°W, 947 m, 16 July 2009, *Johnson 09-109* (BRY); About 7.3 miles up Wildwood Rd (NF-3) from Hwy 36, 40.4514°N, 123.0649°W, 1003 m, 13 July 2016, *Johnson 16-063a* (BRY); South of Hayfork about 3.3 miles along Kingsbury road from jct with Morgan Hill road, 40.5074°N, 123.1469°W, 851 m, 13 July 2016, *Johnson 16-070* (BRY, JEPS); Yuba County, along margin of New York Flat Road, 0.5 miles north from jct with Laporte road (possible waif), 39.4812°N, 121.2606°W, 2255 ft, 18 June 2004, *Johnson 04-150* (BRY).

#### Notes.

*Navarretia aeroides* are mephic when fresh. In 2015, the population first collected by Barbe, Fuller, & Howell in the mountains east of Quincy, California, was found to have been sprayed with 2,4-D (and blue indicator dye), along with *N. propinqua*, in an area designated for ORV use, perhaps having been mistaken for immature thistle. To date, occurrences in the Trinity Mountains can be distinguished morphologically (white corollas with magenta streaking in the throat and white pollen) from occurrences in the Sierra Nevada (blue to light blue corollas and blue pollen), yet we have resisted recognizing this difference at the subspecific level. As in any species with colored corollas, occasional white flowered individuals are observed in the Sierra Nevada among a sea of blue flowered individuals. The paratype of *Gilia atrata* M.E.Jones from Colfax, California [POM-75128 scan!] belongs here.

*Navarretia aeroides* is a smaller-featured plant than either *N. vividior* or *N. modocensis*, though its corolla overlaps in size with *N. vividior*. The more conspicuously glandular inflorescence heads (in side-by-side comparisons) contrasts with all of the other species detailed here, and corolla coloration, fresh and dried, readily distinguishes this taxon from *N. divaricata* and *N. torreyella*.

### Navarretia torreyella

Taxon classificationPlantaeEricalesPolemoniaceae

L.A.Johnson & D.Gowen sp. nov.

urn:lsid:ipni.org:names:60475569-2

Figs 4 , 7

#### Type.

United States of America. California: El Dorado County, Peavine Ridge Road, along left fork (11N55) about 4.7 miles from junction with Icehouse Road, 38.7961°N, 120.4770°W, 1480 m, 26 June 2013, *L.A.Johnson, R.L.Johnson, & A. Yankee 13-230* (holotype BRY! [BRY-619469]; isotypes JEPS! RSA! and to be distributed).

#### Diagnosis.

A species similar to *Navarretia divaricata*, but distinguished by having three equal stigmatic lobes and three fully developed fruit valves, and generally larger corollas with a deep maroon distal tube and throat abruptly transitioning to nearly white or less commonly pink lobes, the lobes drying lighter than the much darker throat and tube.

#### Description.

***Taprooted annual herbs*** to 7(–10) cm tall and 14(–20) cm wide, sometimes larger, generally wider than tall. Primary stem erect, terminating in an inflorescence head 1–2(–3) cm above the cotyledons; generally greatly exceeded by secondary stems, with tertiary, and quaternary stems present on larger plants; higher order branches arise from axils of proximal inflorescence bracts, axils of leaves subtending the primary head, or less commonly, leaves within 1 cm of an inflorescence head; branches ascending to spreading and ± leafless, except for leaves subtending higher order branches or within 1 cm of a head; stem and branches reddish-brown, sparsely minutely glandular pubescent to glabrescent, less often villous, the trichomes generally less than 0.5 mm long; distal-most branches filiform, generally no more than 0.3 mm in diameter. Cotyledons two, linear, entire, united at base. ***Leaves*** somewhat finely stipitate-glandular proximally, less so distally; leaves at the lowermost nodes opposite, linear-filiform, and widened at the point of stem attachment, the proximal nodes often congested with overlapping leaf bases. More distal leaves alternate, entire, or more commonly with 1–3 paired or unpaired linear lateral lobes 1–5 mm long attached along the proximal 3(–15) mm of the leaf, with an elongated, linear terminal segment. ***Inflorescences*** head-like, generally ≤ 10 mm diameter (exclusive of bract tips; ~15 mm with bract tips), mostly less than 15 flowered, villous proximally, obscurely glandular. Inflorescence bracts < 10(–12) mm long, palmatifid to subpalmatifid; outermost 1–2 bracts with a short achlorophyllous base and 2–3 pairs of lateral lobes flanking an elongate terminal lobe, the distal pair of lateral lobes sometimes shorter and reflexed somewhat out of plane relative to the other lobes; bract bases become larger and clasping centripetally with lateral lobes reduced to a single pair departing from near the apex of the bract base flanking the central terminal lobe, all bract lobes chlorophyllous, entire, long tapering acute. Bracts sparsely villous abaxially, more densely villous adaxially and proximally along the lobes just above the rachis, with the distal 1/2 of each lobe more or less glabrous or with a few minute, stipitate glands. ***Flowers*** actinomorphic, ***calyces*** mostly 4.5–7.8 mm long, tube ~ 1.5–2 mm; costae entire, long tapering acute, strongly unequal to subequal with typically two costae longer than the other three; costae narrowing proximally, the shorter ones narrower at base than the intercostal membrane and the longer ones subequal with the membrane; calyx tube achlorophyllous, minutely glandular-puberulent on the intercostal membrane with the costae at least somewhat villous, the trichomes longest along the costae at the junction with the intercostal membrane, the free portion of the costae glabrous to very sparely, obscurely, and minutely glandular distally; intercostal membrane v-shaped at sinus. ***Corolla*** generally ± equal to the calyx costae at anthesis but exceeding the calyx as fruit matures, narrowly funnelform, glabrous, 4.0–6.0(–6.8) mm long, lobes 0.7–1.5 mm long × 0.5–1.0 mm wide, proximal tube white, distal tube and throat maroon, abruptly transitioning to white or whitish to uncommonly pink lobes; tube base expanding and investing the fruit apex. ***Stamen*** filaments unequal, 0.25–1.2 mm long, inserted unequally to subequally 0.2–1.2 mm below corolla sinuses, included in throat to exserted less than half the length of the corolla lobes; pollen white (uncommonly light blue), apertures pantoporate, acolpate; sexine seimitectate, reticulate, heterobrochate. ***Ovary*** three-chambered, stigmatic lobes three, included in to slightly exerted from the corolla throat; capsule ~ 2.5 mm long, dehiscing circumcisally around the base with valves splitting upward. ***Seeds*** generally 2–5 per locule, medium brown, ovoid-angular, mucilaginous when wet. ***Nuclear gene*** loci showing diploid PCR amplification patterns.

**Figure 7.**
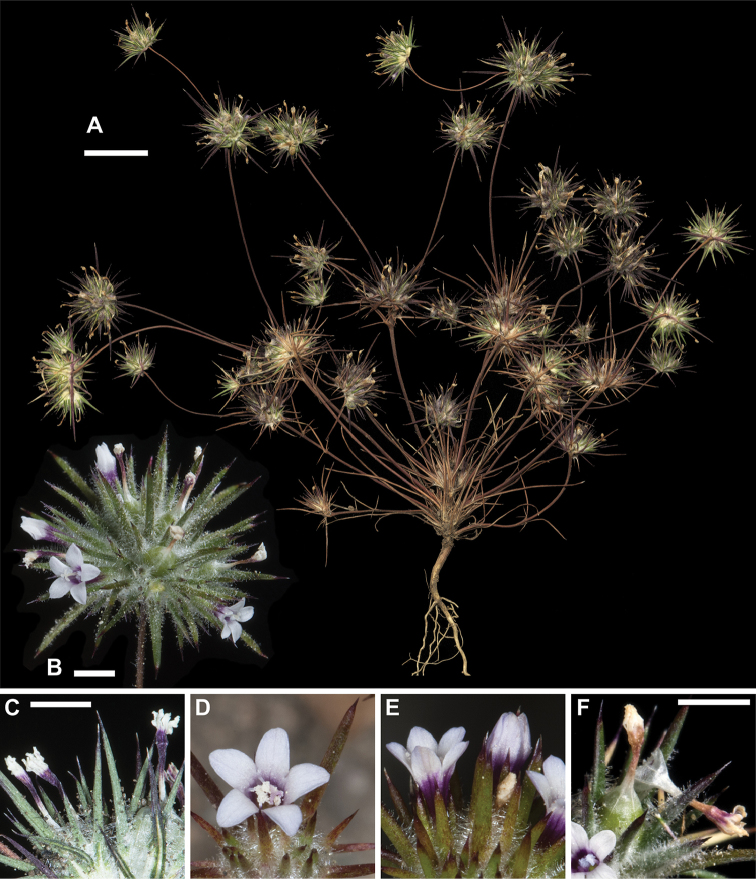
*Navarretia torreyella*. **A** Pressed specimen showing plant habit, scale bar = 1 cm (*Johnson et al, 13-230*) **B** Flowering head in the field, scale bar = 2 mm (*Johnson 16-008*) **C–E** Equivalent magnification, scale bar = 2mm. **C** Pressed flowering head showing coloration of dried flowers (*Johnson 16-008*) **D, E** Fresh flowers showing coloration (*Johnson 13-218*) **F** Fresh flowering head with maturing fruit, showing stretched corolla base that typically clings to fruit through maturity common in all of the species detailed herein, scale bar = 2 mm (*Johnson 16-008*). All photographs by L. A. Johnson and vouchers deposited at BRY.

#### Habitat, distribution, and phenology.

*Navarretia torreyella* occurs on basalt flats, pyroclastic rubble, and clay soils from 1000–2100 meters elevation, in Butte, El Dorado, Nevada, Placer, Plumas, and Tehama Counties, California. Depending on latitude and elevation, it blooms from (May) June–July (September), beginning its flowering a little earlier than *N. divaricata*, and about the same as *N. crystallina*, when these taxa co-occur.

#### Conservation status.

*Navarretia torreyella* has many occurrences throughout its range and is typically abundant when it is encountered. It is a species of Least Concern following IUCN (2012) Red List version 3.1 criteria.

#### Etymology.

In honor of John Torrey for the plant he recognized, before others, as distinct at the species level.

#### Representative specimens examined

**(paratypes). UNITED STATES OF AMERICA. California**: Butte County, about 8 miles east of Feather Falls, 1.5 miles east of Camp 18, yellow pine forest, 1000 meters, 4 June 1982, *Ahart 3533* (CAS, CHSC); About 2 road miles west of Camp Eighteen along La Porte Rd (94), then 0.2 miles north along Frey Ranch Rd, 39.6291°N, 121.1970°W, 1266 m, 10 June 2015, *Johnson & Ahart 15-022* (BRY, JEPS); About 1.2 miles east of Camp Eighteen along Lumpkin Ridge Rd, 39.6277°N, 121.1473°W, 1290 m, 10 June 2015, *Johnson & Ahart 15-028* (BRY, JEPS, RSA); 1.6 miles north of La Porte Road (and 1.8 miles south of south end of Sly Creek Reservoir Dam), T20N R8E S19, 23 June 1980, *Schlising, Ikeda, & Banchero 3765* (CHSC); Lumpkin Ridge, T21N R7E S36, 20 May 1981, *Schlising & Banchero 4059* (CHSC); Near Bull Hill Road, about 3 miles south of Butte Meadows, 1600 m, 16 August 1983, *Ahart 4303* (CHSC); Jackass Flat, Mooreville Ridge, about 1 air mile northwest of the Lost Creek Reservoir Dam, 3800 ft, 20 June 1993, *Ahart 6999* (CHSC); sides of a poor road, east of cow corral, about 200 yards north of the county road, about one mile west of the intersection of county road and Lumpkin Road, about 7 miles northeast of Feather Falls, 39.6294°N, 121.1983°W, 4204 ft, 2 July 2014, *Ahart 19551* (CHSC); Southwest end of Lumpkin Ridge, about 6.75 air miles northeast of town of Feather Falls and 1.3 miles east of the Camp 18 site on topo maps, about 300 ft southeast of USFS Rd 22N27, 2 miles east of its junction with the county road and USFS Rd 22N94 to Fall River, T21N R7E S36 SW1/4 of SE1/4, 4250 ft, 6 June 2005, *Castro 1483* (CHSC); El Dorado County, off road to Ice House Reservoir, 6.4 miles from jct. with Hwy 50, 1596 m, 38.79754°N, 120.40287°W, 30 May 2013, *Johnson 13-218* (BRY, JEPS); Along Park Creek Road, 2.8 miles from junction with Mormon Emigrant Trail, 15 May 2004, *Johnson 04-076* (BRY); Sly Park Vicinity, Park Creek Road off of county road E16, 38.7493°N, 120.4970°W, 1207 m, 8 July 2014, *Johnson 14-143* (BRY); Nevada County, Nevada City, 20–22 June 1912, *Eastwood s.n.* (CAS); Indian Springs Campground, near Cisco, 26 June 1965, *Day 65-146a* (DAV); 1.5 miles west of Cisco Grove, on a western slope above South Fork of Yuba River, 5800 ft, 21 July 1953, *Crampton 1516* (AHUC; mixed collection with *Navarretia divaricata*); Scotts Flat Reservoir on Deer Creek about 5 miles east of Nevada City, 3100 ft, 25 August 1965, *True & J. T. Howell, 2548A* (CAS); Near Grass Valley in red clay in the yellow pine and oak belt, 25 May 1919, *Heller 13197* (CAS, UC); 2 miles west of Grass Valley, gravelly meadow edges, 2350 ft, 5 June 1939, *Rose 39208* (CAS); hills northwest of Grass Valley, 2700 ft, 14 June 1967, *Rose 67134* (BRY); Spur road off north side of Hwy 20, ca. 9–10 miles from I-80 at Yuba Pass, 1565 m, 39.3171°N, 120.7514°W, 3 June 2014, *Johnson* et al. *14-017* (BRY); West of meadow under power lines, about 150 yards west of paved road, southwest of Lake Spaulding, 39.3174°N, 120.6400°W, 5160 ft, 23 July 2005, *Ahart 12185* (CHSC); Small meadow near a small wash between the curve and below the bridge on old highway 80, east of Donner Pass and Donner Summit, 39.6521°N, 120.3169°W, 6767 ft, 31 July 2005, *Ahart 12227* (CHSC); North of Hwy 20 about 8.2 miles west of I-80 ramp at intersection with road 20-16, 39.3171°N, 120.7239°W, 1617 m, 11 July 2016, *Johnson 16-015* (BRY); Placer County, Cisco, 25 June 1910, *Hall 8712* (UC); Rainbow, north of Cisco, 5700 ft, 23 June 1946, *Rose 46201* (UC); Strawberry flat, the Henderson ranch, near Indian Creek, T15N R10E S23 SW1/4 of SW1/4, 3280 ft, 24 May 1969, *Kawahara 870* (CAS); South facing slope of pyroclastic flow overlooking the North Fork of the American River, just off Sawtooth Ridge Road ca. 1.1 mile west of Dawson Spring, 39.2179°N, 120.6227°W, 1670 m, 3 June 2014, *Johnson* et al. *14-028* (BRY, JEPS); Along Forest Hill Road, ca. 0.2 miles northeast of NF-66 (Humbug Canyon Rd.), 39.1605°N, 120.6618°W, 1578 m, 3 June 2014, *Johnson* et al. *14-048* (BRY, JEPS); Off of Sugar Pine Road (NF10) enroute to Sugar Pine Reservoir, ca. 3.6 miles from Forest Hills Road, 1181 m, 39.1239°N, 120.7588°W, 9 June 2015, *Johnson 15-013* (BRY); 50 yards northeast of the small natural lake, on the east side of the paved road to Lake Valley Reservoir, about 1/4 mile east of Yuba Gap and Highway 80, 39.3158°N, 120.6039°W, 5840 ft, 22 July 2002, *Ahart 9896* (CHSC); Plumas County, south of Cascade and Lava Top, access via FS road 21N22YA, 39.6827°N, 121.1665°W, 1402 m, *Johnson & Ahart 15-023* (BRY, JEPS, RSA); Along Lumpkin Ridge-La Porte Rd, ca. 6.9 miles northeast of junction with Golden Trout Crossing, 39.6982°N, 121.0783°W, 1541 m, 10 June 2015, *Johnson & Ahart 15-030* (BRY, JEPS, RSA); About 1/4 mile north of Lumpkin Ridge Road, on Lumpkin Ridge, about 4 air miles northeast of Camp 18, 39.6856°N, 121.1061°W, 5013 ft, 3 July 2006, *Ahart 12905* (CHSC, JEPS); North side of Onion Valley, east of Quincy La Porte Road, about 1 mile northwest of Pilot Peak, T22N R10E S5 SE1/4, 6000 ft, 13 September 1995, *Ahart 7640* (CHSC); About 100 yards north of the paved basalt road, about 3.75 air miles northwest of Tamarack Flat, about 5.75 air miles northwest of Little Grass Valley Reservoir, 39.7615°N, 121.0980°W, 5507 ft, 9 July 2006, *Ahart 12929* (CHSC); West edge of top of Goat Mountain, 0.7 km southeast of the southeast side of Little Grass Valley Reservoir, 39.7181°N, 120.9664°W, 1721 m, 6 July 2011, *Janeway 10497* (CHSC); Tehama County, across fence on southwest side of Hwy 36 about 5.1 miles west of jct with Hwy 32, 40.3278°N, 121.4735°W, 1459 m, 26 June 2017, *Johnson & Johnson 17-042* (BRY).

#### Notes.

*Hall 8712* (UC) describes this collection as faintly malodorous though we have not detected a scent ourselves in this taxon. The *Shelton s.n*. syntype of *Gilia divaricata* Torr. ex Gray belongs here.

When growing with *N. divaricata*, a subtle difference in habit and coloration is discernable, with *N. torreyella* somewhat more spreading, its primary inflorescence head closer to the ground, and the plants overall more anthocyanic (purple tinged); however, as these features can vary in both taxa, they should not be relied upon for determination. Instead, corolla coloration of both fresh and dried flowers as outlined in the diagnosis distinguishes *N. torreyella* from all of the species treated herein. This taxon also has the thinnest, most filiform branches of the species considered here.

##### Key to the taxa formerly treated as *Navarretia divaricata*

**Table:** 

1	Corollas 3.5–5 mm, lobes white or the tips tinged pink to lavender when fresh, drying pink (generally much darker than throat and tube), tube and lower throat yellowish when fresh, similar when dried (sometimes streaked with red); stigmas minute with 2 of 3 lobes fused almost to tips, fruit with 1 (of 3) valves half as wide and lacking a septum	***N. divaricata***
–	Corollas 4–8(+) mm, commonly with blue, lavender, pink, or whitish lobes and similar or darker maroon throat when fresh, drying with lobes and throat blue to purplish, or whitish lobes with reddish-streaked or dark maroon throat; stigmas equally 3-lobed; fruit equally 3-valved, each bearing a septum	**2**
2	Corollas bicolored when fresh with white or less commonly pink lobes abruptly transitioning to a dark maroon throat, drying in similar manner; distal half (or more) of inflorescence bract lobes and calyx costae glabrous or nearly so	***N. torreyella***
–	Corollas variously concolored to bi- or tri-colored when fresh with transition between lobes and throat gradual, bluish, bluish- or pinkish-lavender, or less commonly white, drying in like manner or with darker and/or reddish-streaked throat; distal half of inflorescence bract lobes and calyx costae generally glandular	**3**
3	Largest inflorescence heads exclusive of bract lobes ≤ 10 mm diameter (≤ 15 mm with bract lobes), conspicuously glandular proximally and distally (some villous trichomes present proximally); branches filiform (± 0.3 mm diameter), trichomes mostly < 0.5 mm, sometimes wanting; corollas 4–6 mm, blue (generally with blue pollen) or whitish (with white pollen)	***N. aeroides***
–	Largest inflorescence heads exclusive of bract lobes mostly ≥ 12 mm diameter (≥ 18 mm with bract lobes), ± villous proximally, glandular distally; branches more robust (± 0.5 mm), trichomes commonly > 1 mm; corollas 5–8(+) mm, bluish-lavender or pinkish-lavender, pollen blue or white	**4**
4	Corolla generally 5–7 mm, bluish or bluish-lavender when fresh distally with whitish to yellowish tube, pollen blue; inflorescence heads often more glandular than villous; plants mainly west of the Central Valley in the North Coast and Klamath Ranges (uncommon in Butte County)	***N. vividior***
–	Corolla generally 6–8(+) mm, pinkish-lavender when fresh distally with yellowish tube, pollen white (uncommonly blue); inflorescence heads often more villous than glandular; plants mainly east of the Central Valley in the Modoc Plateau and Cascade Ranges, disjunct in San Benito County	***N. modocensis***

## Methods

Herbarium specimens were examined directly or via high resolution scanned images of herbarium sheets; scans are indicated as such in specimen citations. Specimen collections were examined from UC, JEPS, DAV, AHUC, RSA, POM, CHSC, IDS, BRY, HSC, CAS, ORE, OSC, and WTC. Single or small groups of particular specimens of interest were provided by GH, NDG, NY, RENO, US, and UBC. Specimens examined also included material gathered from our own field work (deposited at BRY, JEPS, or both). Working iteratively between herbarium specimens, field work, and laboratory examination, taxonomic hypotheses were refined following the unified species concept (de Queiroz 2007). For *Navarretia divaricata* only, the distribution map (Fig. 2) was compiled by augmenting examined specimens with data obtained from CDA, CIC, DS, HJAEFB, ID, RM, SOC, SRP, WS, and YM as searchable via the Consortium of California Herbaria (http://ucjeps.berkeley.edu/consortium/) and the Consortium of Pacific Northwest Herbaria (http://pnwherbaria.org). Digital photographs were used to verify determinations of the online records when available, particularly with questionable occurrences in disjunct locations; questionable occurrences were not mapped.

Morphologically, specimens were grouped under the framework of population aggregate analysis/specimen aggregate analysis (Davis and Nixon 1992, Snow et al. 2003). Specimens were examined for qualitative or quantitative features useful for distinguishing among taxa. Digital calipers were used for larger features, while smaller features were measured from digital images taken with an Olympus SZX-12 dissecting microscope using CellSens software (Olympus Soft Imaging Solutions Corp.). All corolla measurements were taken from flowers post-anthesis with expanding to fully mature fruits, after first boiling for ~ 30 seconds and keeping the tissue rehydrated in Pohl’s solution (Pohl 1965).

DNA sequences were examined in the context of exclusivity (Brower 1999), with an emphasis on shared patterns across independent loci given that any single locus may violate this principle in recently diverging groups due to lineage sorting or other recognized biological processes. DNA sequence data was initially obtained from 66 accessions representing 10 species as defined herein. This sampling included 11 accessions of *Navarretia divaricata* and 43 accessions representing the various taxa formerly recognized as Navarretia divaricata subsp. vividior from localities representing the geographic distribution of each taxon. This dataset was simplified a posteriori to include just six accessions of each taxon in the *Navarretia divaricata* complex and a single representative of five related species, selecting accessions that represent the range of genetic variation observed while eliminating much redundancy in the dataset. Focusing on species delimitation rather than phylogenetic reconstruction, the five additional species included in the analyses are morphologically most similar to the focal taxa: *N. crystallina*, *N. filicaulis* (Torr. ex A.Gray) Greene, *N. miwukensis*, *N. peninsularis*, and *N. prolifera*. A concatenated chloroplast matrix was constructed from the 5’ *trnK* intron and 5’ portion of *matK* (Johnson and Soltis 1995, Johnson and Johnson 2006), *trnL–trnL–trnF* intergenic spacer and intron (Taberlet et al. 1991), *trnS–trnG* intergenic spacer (Hamilton 1999), and *rpl16* regions (Small et al. 1998). A matrix from the nuclear ribosomal ITS1, 5.8s, and ITS2 region (White et al. 1990, Porter 1996) was also constructed. For these matrices, amplification primers as described in the papers just cited were used in PCR reactions consisting of 30 cycles of 95°C for 1 min, 52°C for 1 min, and 72°C for 1 min. PCR amplicons were cleaned using PrepEase PCR purification plates (Affymetrix, Santa Clara, California, USA) prior to sequencing with BigDye vs 3.1 (Applied Biosystems/Thermo Fisher Scientific, Waltham, Massachusetts, USA). Sequenced products were cleaned with Sephadex and run on an AB 3730 xl DNA sequencer in the DNA Sequencing Center at Brigham Young University. Three additional matrices from low copy nuclear genes were also constructed: pistillata (Johnson et al. 2012) and two paralogs of isocitrate dehydrogenase (Weese and Johnson 2005; Johnson and Johnson 2006). To simplify data acquisition and cloning, new taxon-specific primers were designed for this study to amplify 600–800 bp segments of these low copy regions that included introns and exons: *PIdv1F* 5’-TGGGTACTCATAGGTTGGTTGA-3’; *Pidv1R* 5’-TGCAAGGAGAGACTTACCTGA-3’; *idhAdv1F* 5’- AGCAATCAAGTGTGCGACAA-3’; *idhAdv1R* 5’- AGCGGCCACTTCTTCTGATA-3’; *idhBdv354F* 5’- CTGCAGATGAAGCTCGTATGG-3’; *idhBdv1082R* 5’- CGTAAGCTGTGGTCATCGAA-3’. For these low copy nuclear regions, a touch-down PCR protocol was employed starting with a 62°C annealing temperature and decreasing 1°C each cycle until reaching 52°C, then repeating 29 additional cycles with a 52°C annealing. When multiple copies were evident from direct sequencing attempts that made chromatograms unreadable (nuclear regions only), PCR amplicons were first purified using PrepEase PCR purification plates, resuspended in 30 µl water, then cloned via TOPO TA for sequencing kits (Invitrogen/ThermoFisher Scientific, Waltham, Massachusetts, USA), with generally eight colonies per cloning reaction subsequently re-amplified via PCR with standard M13 primers and these amplicons sequenced. Sequences have been deposited in GenBank (Appendix 1). Sequence matrices were aligned by eye using AliView (Larsson 2014). Unweighted parsimony analyses using PAUP* 4.0b10 (Swofford 2003) with 10,000 random addition replications, collapsing branches with minimum length of zero, and assessing support with 100,000 bootstrap replications using fast stepwise addition were performed for each matrix and the results from the separate analyses compared by eye.

## Results

**Figure 8.**
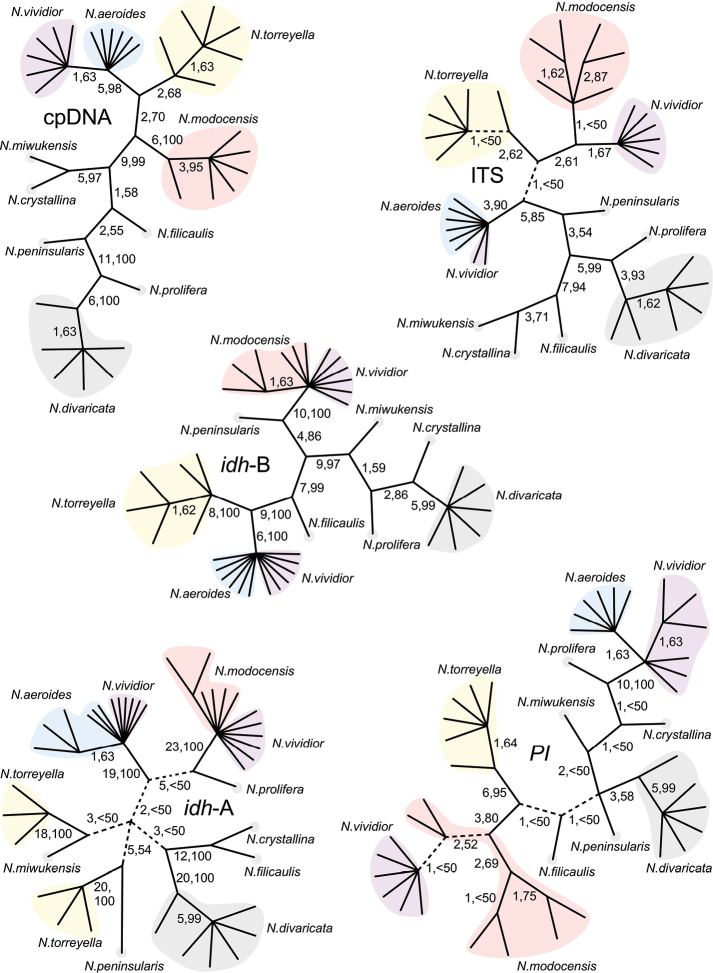
Representative most parsimonious, unrooted trees inferred from analysis of DNA sequence data. Base substitutions are reconstructed along interior branches, followed by bootstrap support values. Shaded regions around terminal branches circumscribe individuals of the same taxon, using colors for *Navarretia vividior*, *N. modocensis*, *N. aeroides*, and *N. torreyella* that correspond with the colors of symbols used in Fig. 4. Branches not found in all most parsimonious topologies for each region are represented by dashed lines. **A** Single topology inferred from concatenated cpDNA sequences **B** One of eight topologies inferred from nrDNA ITS sequences **C** Single topology inferred from nuclear *idh*-B sequences **D** One of 13 topologies inferred from nuclear *idh*-A sequences **E** One of nine topologies inferred from nuclear *PI* sequences.

The concatenated cpDNA matrix consisted of 35 terminals and 4428 nucleotide characters, of which 51 are parsimony informative. Parsimony analysis of this matrix recovered a single topology of 111 steps (Fig. 8A; CI = 0.96; RI = 0.99).

The nrITS sequence matrix consisted of 37 terminals with two populations of *N. vividior* represented by distinct sequences recovered after cloning the original PCR fragments; the remaining four populations of *N. vividior* provided clean reads from direct sequencing of the original PCR products and were not cloned. This matrix was 631 nucleotide characters in length of which 36 were parsimony informative. Parsimony analysis of this matrix recovered 206052 topologies of 66 steps, which narrowed to eight unique topologies after collapsing branches with a minimum length of zero and then filtering to retain only the shorted trees (still of 66 steps; Fig. 8B; CI = 0.82; RI = 0.95).

The *idh*-A, *idh*-B, and *PI* matrices each contained 41 terminals, with each of the six populations of *N. vividior* represented by two distinct sequences recovered following cloning of the original PCR products. Some cloned fragments were clearly chimeras of the two primary sequences with a single break point in each fragment that varied in location among fragments, indicating these chimeras were generated within the PCR reactions. The *idh*-B matrix contained 729 nucleotide characters of which 55 were parsimony informative. Parsimony analysis of this matrix recovered a single topology of 113 steps (Fig. 8C; CI = 0.95; RI = 0.99). The *idh*-A matrix contained 880 nucleotide characters of which 117 were parsimony informative. Parsimony analysis of this matrix recovered 108 topologies of 220 steps, which reduced to 13 after condensing to remove branches with a minimum length of zero and filtering to retain only the shortest topologies (Fig. 8D; CI = 0.87; RI = 0.96). The PI matrix contained 646 nucleotide characters of which 35 were parsimony informative. Parsimony analyses recovered 32 topologies of 83 steps which reduced to nine after condensing to remove branches with a minimum length of zero and filtering to retain only the shortest topologies (Fig. 8E; CI = 0.81; RI = 0.95).

## Discussion

**DNA-based inferences.** In all DNA sequence-based topologies, using markers representing the chloroplast genome and four putatively unlinked nuclear loci (Fig. 8), *Navarretia divaricata* forms an exclusive group well separated from any of the material previously recognized as N. divaricata subsp. vividior. Though sequences representing the latter taxon form an exclusive group in chloroplast and nuclear ITS sequences, this larger exclusive group is divided into smaller exclusive groups that also appear, with two exceptions, as exclusive groups in each of the three low-copy nuclear gene data sets (i.e., *idh*-B, *idh*-A, and *PI*; Fig. 8). The two exceptions involve *N. vividior* as defined herein, and *N. torreyella*. Sampled populations of *N. vividior* all contained two homeologs in the low copy nuclear gene data sets: one that clusters with *N. modocensis*, and one that clusters with *N. aeroides*. This is an expected pattern for a plant of allopolyploid origin. The placement of *N. vividior* in the cpDNA tree (Fig. 8A) solely near *N. aeroides* indicates *N. aeroides* (or its ancestral lineage) was the maternal parent in the formation of *N. vividior*, whereas *N. modocensis* (or its ancestral lineage) was the paternal parent. Gene conversion in the nrITS region in some populations of *N. vividior* has fixed, or nearly fixed this locus in favor of the paternal parent (Fig. 8B), which often happens over time in allopolyploids (e.g., Johnson and Johnson 2006). Allopolyploidy in *N. vividior* is consistent with the chromosome count of 2*n* = 36 recorded by Crampton for plants collected not far from the type locality (*Crampton 494B* [AHUC-038375! UCD-36041!]. In the *idh*-A data set (Fig. 8D), *Navarretia torreyella* failed to form an exclusive group, a pattern that may be interpreted as incomplete lineage sorting in this single locus; the two groups do not correlate with geography and though we did not test it specifically, we suspect that both alleles likely reside within single populations given the geographic proximity of plants from which divergent alleles were sampled. In summary, DNA sequence data support the recognition of five distinct species in what has heretofore been considered a single species with two subspecies.

**Comparative morphology with similar species.** With the exception of *Navarretia filicaulis*, all of the species included in this study share a common branching architecture. They also share features of flower and fruit that distinguish them from other *Navarretia*. The branching architecture consists of a short primary stem terminating in a head-like inflorescence with elongate, more or less leafless secondary stems arising from leaf or outermost bract axils at the base of the inflorescence. Each secondary stem terminates in a head-like inflorescence with tertiary, and even quaternary stems similarly arising from the axils of the outer inflorescence bracts of the higher-order inflorescences, or from leaves that occasionally appear within one cm of an inflorescence (Figs 1, 3, 5–7). When well branched, this pattern gives rise to plants that are typically wider than tall. All species can produce depauperate plants consisting of a single head, and *N. divaricata* sometimes produces plants that are noticeably taller than wide—perhaps when vegetation is dense. Following pollination, it is common in these species for the corolla to detach from the receptacle but remain vested around the upper half of the enlarging fruit, with the corolla base stretched greatly. Spent corollas thus typically adhere to the fruit through maturation rather than being pushed off by the expanding fruit as is common in other *Navarretia*. The fruit detaches circumcisally about its base with the valves separating more or less from the base to the apex. These flower and fruit characteristics are shared by *N. filicaulis* that may even, uncommonly, branch divaricately from the lowermost inflorescence bracts. *Navarretia prolifera* (two subspecies) are distinguished from the species elaborated here by possessing larger flowers with long-exserted stamens. *Navarretia crystallina* and *N. miwukensis*, recently described, vary in their inflorescence architecture by having flowers inserted directly on a common receptacle (Johnson et al. 2016). *Navarretia peninsularis*, at one time placed as a variety of *N. divaricata* (Jepson 1943), is distinguished by having broader terminal lobes to its leaves and bracts and wider calyx costae. Similarity in overall plant size, habit, and diminutive flowers, combined with the absence of analytical study beyond that conducted by Jepson and Bailey (Jepson 1943), have undoubtedly contributed to the view that *N. divaricata* as heretofore defined represents a single species.

**Table 1.** Comparison of features among the species treated in this paper. Uncommon variation is listed in parentheses.

	*N. divaricata*	*N. torreyella*	*N. aeroides*	*N. vividior*	*N. modocensis*
Stem indumentum	Glabrous to sparingly pubescent, glabrescent; uncommonly pubescent	Glabrous to sparingly pubescent, glabrescent; uncommonly pubescent	Glabrous to pubescent, sometimes glabrescent; trichomes generally less than 0.5 mm	Pubescent; trichomes generally ± 1 mm (glabrescent)	Pubescent; trichomes generally ± 1 mm (glabrescent)
Inflorescence indumentum	Villous; obscurely and minutely glandular; distal half of bract and calyx tips ± glabrous to sparsely minutely glandular	Villous; obscurely and minutely glandular; distal half of bract and calyx tips ± glabrous to sparsely minutely glandular	Conspicuously glandular including distal half of bract and calyx tips; some villous trichomes proximally	Overall more glandular than villous; somewhat villous proximally; distal half of bract and calyx tips ± glandular	Overall more villous than glandular; usually conspicuously villous proximally; distal half of bract and calyx tips ± glandular
Calyx tube pubescence (proximal 3/4)	Puberulent and some villous hairs along costae	Puberulent and some villous hairs along costae	Puberulent	Puberulent and some villous hairs along costae	Puberulent and some villous hairs along costae
Corolla coloration	Proximal tube white, distal tube and throat yellow, sometimes red-streaked, transitioning to white lobes suffused with pink or lavender at tips; lobes drying pink	Proximal tube white, distal tube and throat maroon, abruptly transitioning to whitish (pink) lobes; corolla drying similarly	Tube white, lobes and throat bluish; drying bluish purple—or throat and lobes white; throat and lobes drying white to light blue with brownish to magenta streaked distal tube	Tube white proximally, distal tube white or yellow, throat bluish, sometimes streaked with magenta, lobes medium bluish-lavender; similar or darker when dried	Tube white proximally, yellow distally, throat lavender–purple or purplish streaked, lobes light to dark pinkish-lavender; similar or darker changing toward blue when dried
Corolla length	3.5–5 mm	4–6(–6.8) mm	4.2–6 mm	5–7.2 mm	6–8.2 mm
Corolla lobes	0.6–1.0 mm long × 0.4–0.9 mm wide	0.7–1.5 mm long × 0.5–1.0 mm wide	0.75–1.3 mm long × 0.5–0.9(–1.0) mm wide	0.8–1.1(–1.4) mm long × 0.6–0.9(–1.2) mm wide	(1.0–)1.2–1.5(–1.95) mm long × 0.8–1.4 mm wide
Pistil	2 of 3 stigma lobes nearly entirely fused, one valve partially abortive	3 equal lobes, 3 equal valves	3 equal lobes, 3 equal valves	3 equal lobes, 3 equal valves	3 equal lobes, 3 equal valves
Ovules	5–9 (12)/fruit	2–5/locule	4–8/locule	5–7(8)/locule	4–9/locule
Largest inflorescence head diameter (excluding bracts)	≤ 10 mm	≤ 10 mm	≤ 10 mm	≥ 12 mm	≥ 12 mm
Pollen color	White	White (light blue)	Blue or white, matching corolla	Blue (white rarely?)	White (blue)
Scent (fresh)	Indistinct	Indistinct	Skunky	Skunky	Skunky
Ploidy	Diploid	Diploid	Diploid	Allotetraploid	Diploid

**Recognition of near-cryptic species**. Morphology and one’s perception has been the guiding force of species delimitation for centuries. Though some may not admit it, many botanists in the field are sympathetic to Cronquist’s (1978) view that “species are the smallest groups that are consistently and persistently distinct, and distinguishable by ordinary means” (often interpreted as a 10× hand lens). That some plants can distinguish their own pollen versus that from a genetically different individual in the same population demonstrates that organisms can perceive what our eyes cannot. It should not, then, be surprising that different species (i.e., independent evolutionary lineages with distinct trajectories) may be distinguishable genetically by the organisms themselves and biologists with appropriate tools, despite little or no discernable morphological differentiation. This may be inconvenient for field taxonomists, and recognition of such species may be challenging by human eye, yet the taxonomic recognition of biological reality is still merited (Judd et al. 2007; Carolan et al., 2012; Jörger and Schrödl 2013). Withholding taxonomic recognition for cryptic or nearly cryptic species may simplify the placing of a name on an entity by sight, yet also compromises our understanding of diversity via inaccurate estimates of species diversity, superficial understanding of diversification patterns and processes, and inaccurate assessments of species abundance and ecological preferences.

Over several years of field work without observing hybrid swarms or convincing intermediate forms, we suspected that *Navarretia vividior* was a species distinct from *N. divaricata*. In the process of sampling broadly across these species’ ranges, the distinctiveness of *N. torreyella* soon came into focus both morphologically and molecularly. Though similar in many respects, it would be inaccurate to characterize *N. divaricata*, *N. vividior*, and *N. torreyella* as truly cryptic—they can be readily diagnosed via observable morphological differences that can be articulated in a dichotomous key. Either *N. modocensis* or *N. aeroides* could replace *N. vividior* in the above three-way species comparison with little to no editing of the dichotomous key (depending on feature choice). The challenge, morphologically, is in distinguishing between *N. vividior*, *N. modocensis*, and *N. aeroides*. Among these latter three species, *N. aeroides* is most distinctive and may be appropriately considered “near-cryptic”; it is discernable from *N. modocensis* in features relating to the corolla and inflorescence size, and inflorescence glandularity. Nevertheless, the morpho-space distinguishing *N. aeroides* from *N. modocensis* is narrow. Consequently, their allopolyploid derivative, *N. vividior*, has a limited morpho-space for intermediacy by which it can be distinguished from its parents, and in that limited space, it lies closer to *N. modocensis*, its paternal parent, than to *N. aeroides*, but overlaps with both. *Navarretia vividior* and *N. modocensis* are the closest of several morphologically similar species pairs we have investigated (e.g., Johnson et al. 2013, 2016) to being truly cryptic. It is not surprising that the distinctiveness of these taxa has been overlooked in the past, and we recognize that difficulty exists in differentiating these species, particularly in their pressed and dried condition on herbarium sheets.

**Geographic distribution and syntopy.** The taxonomy proposed herein alters our understanding of species distributions and abundance. In its broad distribution that largely encompasses the ranges of the other taxa (compare Figs 2, 4), *Navarretia divaricata* is known to co-occur with *N. modocensis* and *N. torreyella* in many locations, and with *N. aeroides* and *N. vividior* in at least some locations. It can also be found with many other *Navarretia*, including *N. leptalea*, *N. breweri*, *N. capillaris*, *N. linearifolia*, *N. propinqua*, *N. intertexta*, *N. crystallina*, *N. miwukensis*, and *N. prolifera* in portions of its range.

The distribution of *N. vividior* is narrowed to predominantly the North Coast Range, with the exception of apparent occurrences in the foothills of the northern Sierra Nevada/southern Cascade Range in Butte County, California where it may co-occur with *N. modocensis* (Fig. 4). In Trinity County, California, it co-occurs with *N. aeroides* in at least two locations and at times with *N. divaricata. W*e have also collected *N. vividior* growing with *N. atractyloides*, *N. mellita*, *N. squarrosa*, *N. intertexta*, *N. lecucocephala*, *N. subuligera*, and *N. linearifolia*.

*Navarretia modocensis*’ distribution extends beyond the Modoc plateau (Fig. 4). It overlaps with a portion of the range of *N. torreyella* (Fig. 4), though we have not found them co-occurring (both have been collected in the Butte Meadows area). We also have yet to find *N. modocensis* co-occurring with *N. aeroides* or *N. vividior*, though such locations might exist in Butte County, California. We have collected *N. modocensis* with *N. atractyloides*, *N. breweri*, *N. divaricata*, *N. filicaulis*, *N. intertexta*, *N. propinqua*, *N. subuligera*, *N. sinistra*, and *N. linearifolia* in the principle portion of its range, and the disjunct occurrence in San Benito county was co-occurring with *N. mellita*.

The distribution of *Navarretia aeroides* covers considerable geographic area (Fig. 4), yet it has been seldom collected, with over half of the occurrences known only from historical collections over 60 years old. Occurrences appear to be highly fragmented and localized. Awareness of this taxon will undoubtedly aid efforts to better define its abundance. All occurrences in the North Coast Range that we are aware of have been pale (whitish) flowered individuals with white pollen that contrast sharply with the larger and hairier-headed *N. vividior* with its blue flowers and blue pollen when the two co-occur. Besides *N. vividior*, we have observed *N. aeroides* co-occurring with *N. atractyloides* and *N. intertexta* in the North Coast Range, and with *N. divaricata*, *N. filicaulis*, *N. propinqua*, N. prolifera subsp. lutea, *N. miwukensis*, N. leptalea subsp. leptalea, in addition to growing near *N. torreyella* in the Sierra Nevada. The latter occurrence, observed in July, found *N. aeroides* on one side of a dirt 4WD road in bloom with *N. divaricata* and N. prolifera subsp. lutea, while *N. torreyella* occurred completely senesced in monoculture on the opposite side of the road.

Though *N. torreylla* has the smallest geographic distribution of the species considered here (Fig. 4), it occurs abundantly both in number of individuals per occurrence and number of occurrences within its range. It grows intermixed with *N. divaricata* in many locations and herbarium sheets of mixed collections originally determined as either N. divaricata subsp. divaricata or N. divaricata subsp. vividior exist. *Navarretia propinqua*, N. prolifera subsp. lutea, *N. crystallina*, and N. leptalea subsp. leptalea also co-occur at times, and at least one observation exists of it growing near *N. aeroides* as described above.

**Butte County, California collections**. Several collections housed at CHSC are intriguing because they compare favorably with either *N. modocensis* or *N. vividior*—and *N. vividior* is otherwise found only on the west side of the central valley. Occurring at somewhat lower elevations, in a hotter environment in the transition between the northern Sierra Nevada and southern Cascade Range, some of these occurrences flower earlier than typical for both *N. modocensis* and *N. vividior*. Because of their parent/offspring relationship and the difficulty at times in distinguishing these two species on herbarium sheets, we have attempted to relocate these populations over the past several years with limited success because of drought, private property access, and possibly invasive species density. For example, in 1980, *Schlising & Azevedo 3699* collected *N. modocensis* at an ecotone that we visited in 2015; the area was extremely dry and though *N. filicaulis* was found in abundance, a thorough search found no sign of *N. modocensis*. A return visit in 2017, following closer to normal precipitation in the preceding Fall and Winter months (http://www.usclimatedata.com) found the area to be lusher, and both *N. modocensis* and *N. filicaulis* present in abundance (*Johnson 17-027*, BRY). On the other hand, no sign of *N. modocensis* could be found at the site of *Jokerst* et al. *462* in 2017, yet the species was described as common when collected in 1979 growing with *N. tagetina*, which was found in abundance in 2017. *Oswald 578* documents what we have determined to be *N. vividior* along the north rim of Upper Bidwell Park in 1983 following a burn. In 2017, *N. intertexta*, *N. pubescens*, *N. tagetina*, and *N. viscidula* were observed in abundance hiking the entire length of the north rim trail without trace of *N. vividior*, but many areas of possible habitat were covered with dense, near monotypic stands of *Centaurea solstitialis* L. growing through considerable plant litter from previous years. Efforts to contact land-owners for property access permission may enable occurrences that have not been recollected (to our knowledge) in the last 30–40 years to be relocated and assessed to see if any represent mixed populations along Cohasset Road (*Schlising 3435, Ikeda 383*, *Oswald 1999*), near Paradise (*Ahart 1906*), and along Ponderosa Way (*Taylor 1393*, *Oswald 3773*).

### Additional notes on the syntypes for *Gilia divaricata* Torr. ex Gray

*Rattan s.n.*—Volmey Rattan, a notable student of the California and Pacific Coast flora and accomplished teacher (Jepson 1928) contributed two collections to the California Geological Survey between 1860–1867 of what is now considered *Navarretia prolifera*. Though the syntype at NY (mounted on the same sheet as the syntypes *Mr. Shelton s.n.* and *Mrs. Davis 49*) lacks a collector’s number, Rattan did number his collections. *Rattan 7*, collected at Reservoir Hill, Placerville (some labels read “San Francisco” instead of Placerville, an erroneous later labeling by an unknown hand) is equated to *Bolander 6422*, or simply labeled 6422. The number 6422 is Henry N. Bolander’s field book number. Bolander served as the state botanist for California for several years beginning in 1864 and played a prominent role in documenting plants as part of the California Geologic Survey (Jepson 1898). In his field notes, Bolander records, “6417–6462 Mr. Rattan’s collection It was my intention to renumber his plants; but was obliged to give it up being pressed too much by work” (unpublished field notes courtesy of GH). *Rattan 45* (= *Bolander 6436*) is from an uncertain locality. These specimens were apparently sent first to US, then distributed from there, with some material reaching A. Gray. *Rattan 7* compares favorably with N. prolifera subsp. lutea (yellow lobed), while *Rattan 45* compares favorably with N. prolifera subsp. prolifera (blue to purple lobed). Though subsp. lutea can dry with bluish lobes, fresh material is distinct. The syntype material at NY! [NY-00336830] appears to have one of each of Rattan’s collections, with a note in Gray’s hand “Fls. yellow ? & purple”. The material at K [K-000769074] also has one of each, flanking *Bolander 4098*, with a note by Gray indicating “…also Rattan”. We have located isosyntypes (labeled variously as indicated above) at UC (scan!), DS (scan!), KEW (scan!), NY!, and US (scan!).

*Torrey 302*—In 1865, John Torrey took a joint business and pleasure trip to California to see living specimens of plants he was familiar with only as pressed specimens (Robbins 1968). Among the plants he collected on this trip, we located two sheets [GH-00274982 (scan!) & NY-00336832 (scan!)] of *Torrey 302*, each with one mounted specimen of this collection, though as many as five specimens may have been collected with the intent of one being kept for his own herbarium, one sent to Asa Gray, one for the Smithsonian, one (perhaps) for Kew, and the fifth to be distributed as determined later (Robbins 1968). Both sheets have printed labels with the location and date provided by Torrey. The specimen now at NY was originally part of Torrey’s Herbarium and in his hand-writing reads simply, “*Navarretia*”, whereas the specimen at GH was left blank by Torrey. Both specimens, in purple ink and Gray’s handwriting, are annotated as *Gilia divaricata* Torr. It is possible that Torrey did not consider this plant conspecific with the syntype that follows, which he also had in his possession.

*Shelton s.n.*—Christopher Shelton was a well-known local botanist credited with bringing the first honey-bees to California. Though his collection is undated and lacking a location, it must have been collected in 1852 or earlier because he was killed aboard the steamboat Jenny Lind when it exploded in San Francisco Bay in April 1853 (Ewan 1981). The handwriting denoting the name of this specimen on the type sheet is attributed to Torrey; the indication that the specimen is an isotype was likely added by Cronquist. The lower right corner of the sheet bears the writing “California Mr. Shelton” also in Torrey’s hand, with a second note of uncertain authorship written below “Also in Herb. Gray – marked ‘Sierra Nevada, Calif. Rattan.’” We interpret this latter note, not in Torrey’s hand, as indicating that Rattan’s collection (mounted on the same sheet) is to be found in GH, marked Sierra Nevada (though we have not located such a specimen). The common interpretation has been that this second note is referring to the province of Shelton’s collection—an interpretation that can be disputed given the reference to Rattan and the uncertain authorship. Nevertheless, as noted above, the morphology of Shelton’s collection matches plants known only from the Sierra Nevada. How and when this specimen came into Torrey’s possession before being passed on to Gray is unknown to us.

*Bolander 4908*—Henry Bolander was an important botanist of the California flora in the mid 1800s. He collected this specimen between 14 June and 17 July 1866 in Yosemite Valley as part of his work with the California Geologic Survey, and in the company of Clarence King’s survey of Yosemite (unpublished field notes courtesy of GH; Jepson 1898). This collection was widely distributed with specimens located at YALE (scan!), US (scan!), UC!, GH (scan!), NY!, K (scan!).

*Mrs. Davis 49*—Nancy Jane Davis was principal of the Birmingham School in Pennsylvania for 60 years (Jepson 1934), and collected plants primarily in the eastern United States. This specimen was collected in July 1867 from Nevada County, on the first of three visits to California. The appellation “Mrs.” was applied by Gray, who received many of her collections; the original label for the syntype reads, “N. J. Davis,” while other collections of hers read, “Miss N. J. Davis.”

## Supplementary Material

XML Treatment for Navarretia divaricata

XML Treatment for Navarretia vividior

XML Treatment for Navarretia modocensis

XML Treatment for Navarretia aeroides

XML Treatment for Navarretia torreyella

## Appendix I

Voucher specimens and GenBank accession numbers used in DNA sequence analyses. Information is presented alphabetically by species in two groups (non-focal species, focal species) in the following order: ***Taxon***: collector(s) & collection number, nrITS, *PI*, *idh*-A, *idh*-B, *trnK*, *matK*, *trnL*, *trnS*, *rpl16*. For the tetraploid *N. vividior*, where two homeologs were obtained from nuclear regions, the accession numbers for each homeolog are included within parentheses. GenBank numbers prefixed with MG are new to this study; those prefixed with KX are from Johnson et al. (2016). Those prefixed with HQ are from an unpublished thesis from the first author’s lab (Green 2010). All vouchers are deposited at BRY.

**Non-focal species. *Navarretia crystallina* L.A.Johnson & D.Gowen**: *Johnson* et al. *14-030*, KX017878, MG022355, MG022227, MG022268, KX017924, KX017856, KX017946, KX017968, KX017902. ***Navarretia filicaulis* (Torr. ex A.Gray) Greene**: *Johnson 04-068*, HQ116860, HQ116921, MG022228, MG022269, HQ117005, HQ116961, HQ117085, HQ117046, KX017897. ***Navarretia miwukensis* D.Gowen & L.A.Johnson**: *Johnson* et al. *14-067*, KX017881, MG022356, MG022229, MG022270, KX017927, KX017859, KX017949, KX017971, KX017905. ***Navarretia peninsularis* Greene**: *Johnson & Zhang 05-116*, HQ116864, HQ116926, MG022230, MG022271, HQ117010, HQ116965, HQ117089, HQ117050, MG022182. ***Navarretia prolifera* Greene**: *Johnson 09-065*, KX017873, MG022357, MG022231, MG022272, KX017919, KX017851, KX017941, KX017963, KX017896.

**Focal species. *Navarretia aeroides* L.A.Johnson & D.Gowen**: *Johnson 09-109*, MG022309, MG022358, MG022232, MG022273, MG022205, MG022333, MG022394, MG022416, MG022183. *Johnson 14-142*, MG022310, MG022359, MG022233, MG022274, MG022206, MG022334, MG022395, MG022417, MG022184. *Johnson 15-005*, MG022311, MG022360, MG022234, MG022275, MG022207, MG022335, MG022396, MG022418, MG022185. *Johnson 15-065*, MG022312, MG022361, MG022235, MG022276, MG022208, MG022336, MG022397, MG022419, MG022186. *Johnson 16-063A*, MG022313, MG022362, MG022236, MG022277, MG022209, MG022337, MG022398, MG022420, MG022187. *Gowen 1303*, MG022314, MG022363, MG022237, MG022278, MG022210, MG022338, MG022399, MG022421, MG022188. ***Navarretia divaricata* Greene**: *Johnson & Johnson 94-049*, KX017870, MG022364, MG022238, MG022279, KX017916, KX017848, KX017938, KX017960, KX017892. *Johnson 94-065*, KX017865, MG022365, MG022239, MG022280, KX017911, KX017843, KX017933, KX017955, KX017887. *Johnson 04-140*, KX017866, MG022366, MG022240, MG022281, KX017912, KX017844, KX017934, KX017956, KX017888. *Johnson 11-029*, KX017867, MG022367, MG022241, MG022282, KX017913, KX017845, KX017935, KX017957, KX017889. *Johnson 13-219*, KX017868, MG022368, MG022242, MG022283, KX017914, KX017846, KX017936, KX017958, KX017890. *Porter & Machen 13772*, KX017869, MG022369, MG022243, MG022284, KX017915, KX017847, KX017937, KX017959, KX017891. ***Navarretia modocensis* L.A.Johnson & D.Gowen**: *Johnson 95-053*, MG022315, MG022370, MG022244, MG022285, MG022211, MG022339, MG022400, MG022422, MG022189. *Johnson 04-130*, MG022316, MG022371, MG022245, MG022286, MG022212, MG022340, MG022401, MG022423, MG022190. *Johnson 14-170*, MG022317, MG022372, MG022246, MG022287, MG022213, MG022341, MG022402, MG022424, MG022191. *Johnson 15-057*, MG022318, MG022373, MG022247, MG022288, MG022214, MG022342, MG022403, MG022425, MG022192. *Johnson 16-086*, MG022319, MG022374, MG022248, MG022289, MG022215, MG022343, MG022404, MG022426, MG022193. *O’dell s.n.*, MG022320, MG022375, MG022249, MG022290, MG022216, MG022344, MG022405, MG022427, MG022194. ***Navarretia torreyella* L.A.Johnson & D.Gowen**: *Johnson, Johnson, & Yankee 13-230*, MG022321, MG022376, MG022250, MG022291, MG022217, MG022345, MG022406, MG022428, MG022195. *Johnson* et al. *14-028*, MG022322, MG022377, MG022251, MG022292, MG022218, MG022346, MG022407, MG022429, MG022196. *Johnson 14-143*, MG022323, MG022378, MG022252, MG022293, MG022219, MG022347, MG022408, MG022430, MG022197. *Johnson & Ahart 15-022*, MG022324, MG022379, MG022253, MG022294, MG022220, MG022348, MG022409, MG022431, MG022198. *Johnson & Ahart 15-028*, MG022325, MG022380, MG022254, MG022295, MG022221, MG022349, MG022410, MG022432, MG022199. *Johnson 16-008*, MG022326, MG022381, MG022255, MG022296, MG022222, MG022350, MG022411, MG022433, MG022200. ***Navarretia vividior* (Jeps. & V.L.Bailey) L.A.Johnson & D.Gowen**: *Johnson 04-032*, (MG022327, MG022328), (MG022382, MG022383), (MG022256, MG022257), (MG022297, MG022298), MG022223, MG022351, MG022412, MG022434, MG022201. *Johnson 05-021*, (MG022329, MG022330), (MG022384, MG022385), (MG022258, MG022259), (MG022299, MG022300), MG022224, MG022352, MG022413, MG022435, MG022202. *Johnson & Gowen 09-049*, KX017885, (MG022386, MG022387), (MG022260, MG022261), (MG022301, MG022302), KX017931, KX017863, KX017953, KX017975, KX017909. *Johnson 16-058*, MG022331, (MG022388, MG022389), (MG022262, MG022263), (MG022303, MG022304), MG022225, MG022353, MG022414, MG022436, MG022203. *Johnson 16-062*, MG022332, (MG022390, MG022391), (MG022264, MG022265), (MG022305, MG022306), MG022226, MG022354, MG022415, MG022437, MG022204. *Gowen 452*, KX017886, (MG022392, MG022393), (MG022266, MG022267), (MG022307, MG022308), KX017932, KX017864, KX017954, KX017976, KX017910.
